# Molecular processes underlying synergistic linuron mineralization in a triple‐species bacterial consortium biofilm revealed by differential transcriptomics

**DOI:** 10.1002/mbo3.559

**Published:** 2018-01-03

**Authors:** Pieter Albers, Bram Weytjens, René De Mot, Kathleen Marchal, Dirk Springael

**Affiliations:** ^1^ Division of Soil and Water Management Department of Earth and Environmental Sciences KU Leuven Leuven Belgium; ^2^ Department of Microbial and Molecular Systems Centre of Microbial and Plant Genetics KU Leuven Leuven Belgium; ^3^ Department of Plant Biotechnology and Bioinformatics Ghent University Gent Belgium; ^4^ Department of Information Technology, IDLab, IMEC Ghent University Gent Belgium; ^5^ Bioinformatics Institute Ghent Gent Belgium

**Keywords:** biodegradation, consortium, cooperation, differential transcriptomics, linuron, synergistic interactions

## Abstract

The proteobacteria *Variovorax* sp. WDL1, *Comamonas testosteroni* WDL7, and *Hyphomicrobium sulfonivorans* WDL6 compose a triple‐species consortium that synergistically degrades and grows on the phenylurea herbicide linuron. To acquire a better insight into the interactions between the consortium members and the underlying molecular mechanisms, we compared the transcriptomes of the key biodegrading strains WDL7 and WDL1 grown as biofilms in either isolation or consortium conditions by differential RNAseq analysis. Differentially expressed pathways and cellular systems were inferred using the network‐based algorithm PheNetic. Coculturing affected mainly metabolism in WDL1. Significantly enhanced expression of *hylA* encoding linuron hydrolase was observed. Moreover, differential expression of several pathways involved in carbohydrate, amino acid, nitrogen, and sulfur metabolism was observed indicating that WDL1 gains carbon and energy from linuron indirectly by consuming excretion products from WDL7 and/or WDL6. Moreover, in consortium conditions, WDL1 showed a pronounced stress response and overexpression of cell to cell interaction systems such as quorum sensing, contact‐dependent inhibition, and Type VI secretion. Since the latter two systems can mediate interference competition, it prompts the question if synergistic linuron degradation is the result of true adaptive cooperation or rather a facultative interaction between bacteria that coincidentally occupy complementary metabolic niches.

## 1 INTRODUCTION

Mineralization of organic xenobiotic compounds is often performed by microbial consortia by means of metabolic association in which one organism in the consortium converts the organic xenobiotic into metabolites that are degraded by other consortium members (Horemans, Albers, & Springael, 2016). Further degradation of downstream metabolites can enhance the initial degradation step resulting in an overall increased efficiency of mineralization of the organic xenobiotic in which case the metabolic association between consortium members is called synergistic. Members of organic xenobiotic degrading consortia are often heterotrophic organisms that also feed singly on compounds other than the organic xenobiotic. Therefore, it is not always clear whether metabolic association between consortium members is a beneficial interaction that has evolved by cooperative adaptation, at least in part because of this purpose (West, Griffin, & Gardner, 2007), or rather represents a facultative interaction between species that coincidentally occupy complementary metabolic niches. In fact, the evolution of cooperative traits is estimated to be rare in the microbial world (Foster & Bell, 2012). The rare examples of cooperation among bacteria show that the involved bacteria typically have developed specialized molecular mechanisms necessary for synergistic functioning of the consortium, such as coaggregration or bacterial signaling (Rickard et al., 2006; Shimoyama, Kato, Ishii, & Watanabe, 2009). The identification of such molecular mechanisms can therefore be indicative of the evolution of cooperation between bacterial consortium members.

A consortium that synergistically degrades an organic xenobiotic compound has been described for mineralization of the widely used phenylurea herbicide linuron (Dejonghe et al., 2003). The consortium was enriched from an orchard soil with a history of linuron treatment and originally consisted of five to six species with functional redundancy. The consortium can be reduced to three partners, that is, strains *Variovorax* sp. WDL1, *Comamonas testosteroni* WDL7, and *Hyphomicrobium sulfonivorans* WDL6, that together provide all steps of the catabolic pathway for linuron mineralization (Dejonghe et al., 2003). Metabolic association, that is, the exchange of linuron metabolites, has been identified as the major driver for synergistic linuron degradation by the consortium. *Variovorax* sp. WDL1 hydrolyzes linuron into 3,4‐dichloroaniline (3,4‐DCA) and *N*,*O*‐dimethylhydroxylamine (*N*,*O‐*DMHA) using the phenylurea hydrolase HylA (Bers et al., 2013; Dejonghe et al., 2003). Although WDL1 contains *dca* and *ccd* clusters encoding for the further degradation of 3,4‐DCA to 3‐oxoadipate via a chlorocatechol intermediate, 3,4‐DCA is degraded inefficiently by WDL1. Instead, 3,4‐DCA and *N*,*O*‐DMHA are excreted by WDL1 and used as carbon and energy source by WDL7 and WDL6, respectively (Dejonghe et al., 2003). When the consortium is grown as a biofilm on linuron as the sole carbon, nitrogen, and energy source, the removal of 3,4‐DCA by WDL7 increases the rate of linuron hydrolysis by strain WDL1, whereas *N*,*O*‐DMHA degradation by WDL6 has no effect on linuron hydrolysis. Therefore, WDL7 is considered to act as a mutualistic partner of WDL1, whereas WDL6 is suggested to have a rather commensal role (Breugelmans et al., 2008). The metabolic interaction between the three strains is further reflected in their close colocalization when grown as biofilms on linuron (Breugelmans et al., 2008). Conservation of species composition of linuron‐degrading consortia as suggested by the isolation of such consortia from geographically separated and physicochemically different soils (Breugelmans, D'Huys, De Mot, & Springael, 2007) and from molecular ecology studies (Breugelmans et al., 2007; Dealtry et al., 2016; Horemans, Bers, et al., 2016) underlines the ecological relevance of this multispecies bacterial organization. Although metabolic association during linuron degradation appears the major driving force of the consortium composition and functionality, we do not know whether other interactions underlie the synergistic degradation of linuron. For instance, it is not yet clear which carbon source(s) support the growth of the primary linuron degrader strain WDL1 in the consortium. Neither do we know whether contact‐dependent and/or ‐independent mechanisms drive the establishment of synergistic interactions between the consortium members, and which could be indicative of true cooperative adaptation (West et al., 2007).

Differential transcriptomics using next‐generation Illumina transcript‐sequencing technology (RNAseq), in which global gene expression is compared between strains when grown in consortium and in isolation, has been recently successfully used to identify candidate genes relevant for interspecies interactions in both artificially composed consortia (Garbeva, Silby, Raaijmakers, Levy, & Boer, 2011) and in syntrophic consortia (Beliaev et al., 2014; Perez et al., 2015; Rosenthal, Matson, Eldar, & Leadbetter, 2011; Tai, Paulsen, Phillippy, Johnson, & Palenik, 2009). In its first application to scrutinize an organic xenobiotic‐degrading consortium, we used differential RNAseq to identify molecular mechanisms mediating synergistic interactions between the members of the linuron‐degrading consortium consisting of *Variovorax* sp. WDL1, *C. testosteroni* WDL7, and *H*. *sulfonivorans* WDL6, grown as biofilms. Focus was on mutualistic partners WDL1 and WDL7 for which gene expression was compared between consortium and monoculture biofilms fed with linuron or 3,4‐DCA as the sole carbon and energy source.

## 2 MATERIALS AND METHODS

### 2.1 Bacteria, media, and biofilm growth conditions

Biofilms were grown at 25°C in a continuous flow chamber system (^©^BioCentrum DTU, Denmark) as described by Breugelmans et al. (2008). *Variovorax* sp. WDL1 (LMG 27260), *C. testosteroni* WDL7 (LMG 27261), and *H. sulfonivorans* WDL6 (LMG 27262) cell suspensions for inoculation were prepared as described previously (Horemans, Smolders, & Springael, 2013). Consortium biofilms and WDL1 monoculture biofilms were grown on nitrogen‐containing mineral medium (MMO; Boon, Goris, De Vos, Verstraete, & Top, 2001) supplemented with 20 mg/L linuron. Monoculture biofilms of WDL7 were grown on MMO supplemented with 14 mg/L 3,4‐DCA. Consortium biofilms as well as WDL1 and WDL7 monoculture biofilms were grown in triplicate. Triplicate noninoculated control systems for abiotic removal of linuron/3,4‐DCA were operated in parallel. At regular time intervals, 1 ml effluent samples were taken, centrifuged at 10,000 *g* for 5 min, and the supernatant stored at −20°C prior to HPLC analysis of linuron and 3,4‐DCA concentrations as described previously (Horemans, Hofkens, Smolders, & Springael, 2014). The theoretical maximal accumulated concentration of 3,4‐DCA in linuron‐fed biofilms was calculated as the molar equivalent of the linuron influent concentration, that is, the concentration of 3,4‐DCA in case all linuron is converted into 3,4‐DCA. Consortium and WDL1/WDL7 monoculture biofilms were harvested after 2 weeks of steady‐state linuron and/or 3,4‐DCA degradation. In all experiments, linuron and 3,4‐DCA PESTANAL analytical standards (99.9%; Sigma‐Aldrich, Belgium) were used.

### 2.2 Draft genome sequence of the consortium members

Cell cultures of strains WDL1, WDL6, and WDL7 for sequencing were prepared as follows. WDL1 was plated from a cryoculture and grown on R2A (Reasoner & Geldreich, 1985) supplemented with 20 mg/L linuron for 4 days at 25°C. WDL6 was plated and grown on MMO agar plates supplemented with 1% (vol/vol) methanol (Boon et al., 2001) for 6 days at 25°C; WDL7 was plated and grown on Luria–Bertani (LB) agar (Sambrook & Russell, 2001) overnight at 25°C. A smear of colonies of WDL1 and WDL6 and a colony of WDL7 was inoculated in R2B supplemented with 20 mg/L linuron, in liquid MMO supplemented with 1% (vol/vol) methanol, and in LB, respectively, and cultures were grown for 4 days, 4 days, and overnight, respectively, until exponential phase. Genomic DNA was extracted from the cultures using the Puregene Core kit A (Qiagen) following the manufacturer's instructions. A paired‐end library (90 bp reads with an insert length of 500 bp) of WDL6 genomic DNA was sequenced by BGI Tech Solutions (Hong Kong) using the Illumina Hi‐seq platform resulting into 418 MB of sequence information. Sequencing of the WDL7 and WDL1 genomes was performed by Baseclear (the Netherlands) using Illumina Hi‐seq based on 75 cycle paired‐ended reads with an insert length of 400 bp resulting in a total of 751 MB of WDL7 and 300 MB of WDL1 genomic sequence information. Nucleotides with a PHRED quality score <20 were trimmed from the end of raw reads and trimmed reads with a length <10 were discarded using the FASTX‐Toolkit‐0.0.12 software (http://hannonlab.cshl.edu/fastx_toolkit/). Draft genome sequences were obtained by assembling the trimmed paired‐end reads into contigs using Velvet (version 1.2.01) with an optimized k‐mer length of 41, 51, and 41 for WDL1, WDL6, and WDL7, respectively, and a minimal contig length of 100 bp (Zerbino & Birney, 2008). The draft genomes were annotated using the web‐based RAST server (Aziz et al., 2008). The PHAST web server was used to identify prophages in the bacterial genomes (Zhou, Liang, Lynch, Dennis, & Wishart, 2011). Undetected or erroneously annotated linuron catabolic genes were identified and reannotated manually. The details of the draft genome assemblies are summarized in Table S3.

### 2.3 RNA extraction, library preparation, and sequencing

Biofilm cells were flushed out of the flow chambers by injecting and pipetting up and down (10 times) 1 mL of ice‐cold RNase‐stop solution (5% water‐saturated phenol/95% ethanol mixture diluted [1:5 v/v] in deionized water). The cells were snap frozen in liquid nitrogen and stored at −80°C. After thawing on ice, biofilm biomass was pelleted by centrifugation at 15,000 *g* for 2 min at 4°C and total RNA was extracted using the SV total RNA isolation kit (Promega) with minor modifications. Briefly, the pellet was resuspended in 100 μl lysis buffer containing 50 mg/ml chicken egg white lysozyme (Sigma‐Aldrich) and incubated for 4 min at room temperature. Further extraction was performed according to the manufacturer's instructions, except that after washing and dissolving in RNase‐free water, the nucleic acid extract was treated twice with TURBO DNase using a TURBO DNA‐free kit (Ambion). Lack of DNA contamination was verified by conventional PCR targeting the 16S rRNA gene of WDL1 and WDL7 as described in Data S1. rRNA was removed from the RNA extracts through subtractive hybridization using the MICROBExpress^™^ kit (Ambion) according to the manufacturer's protocol. The rRNA‐depleted RNA was dissolved in nuclease‐free water. RNA quality and concentration were estimated by spectrophotometry (NanoDrop) and gel electrophoresis (Experion, Bio‐Rad) before and after rRNA depletion.

Of rRNA‐depleted RNA, 1.0–1.5 ng was used to construct RNAseq libraries using the ScriptSeq^™^v2 RNAseq Library Preparation kit (Epicentre) for three consortium biofilms, three WDL7 monoculture biofilms, and one WDL1 monoculture biofilm. Only one WDL1 biofilm RNA extract was used since insufficient RNA was extracted from the other two WDL1 monoculture biofilm samples. Index reads were added to the libraries using the Scriptseq Index PCR Primers (Epicentre) and the barcoded libraries were PCR amplified according to the manufacturer's instructions. The Agencourt AMPure XP system (Beckman Coulter) was used to purify both the 3′‐terminal‐tagged cDNA and the final RNAseq library. Size distribution of the libraries was assessed by agarose gel electrophoresis. Library cDNA concentrations were quantified using a Qubit^™^ fluorometer (Invitrogen) and adjusted to 2 nM. Since WDL6 represents about 10% of the total biovolume in consortium samples (Breugelmans et al., 2008), the seven equimolar consortium and monoculture libraries were multiplexed in a 5:1 volume ratio prior to sequencing to obtain sufficient transcript reads of all strains in the consortium samples. Hundred cycle paired‐ended reads were obtained by Illumina Hiseq sequencing at the Genomics Core UZ Leuven facility (KU Leuven).

### 2.4 Determination of differential gene expression values

Sequences were trimmed to only retain bases with a PHRED quality score of at least 30 near their ends using Trimmomatic version 0.32. For every replicate separately, the trimmed reads were aligned against a triple‐species reference genome sequence consisting of the compiled genome sequences of strains WDL1, WDL6, and WDL7. Bowtie 2 version 2.2.6 was used for the alignment of paired‐end data. Read pairs aligning at different positions in the triple‐species reference genome with identical mapping scores were classified as ambiguous and discarded. When a read pair mapped only one time discordantly, that is, only one of the reads from the pair mapped uniquely to the triple‐species reference genome, that read was considered as mapped and retained. Simply discarding ambiguous reads would reduce the estimated expression level of genes with similar sequences and hence result in false expression rates (Ilut et al., 2012). Therefore, we adapted a method developed by Ilut et al. (2012), in which for each gene a scaling factor is calculated that adjusts the expression levels inferred from read counts to account for the likelihood of undercounting expression of genes with similar sequences. For both WDL1 and WDL7, only 0.3% of the genes had a scaling factor different from 1, meaning this adjustment has an insignificant effect on the analysis. The calculated scaling factors for all genes in WDL1 and WDL7 can be found in the Supporting Information (Table S4). To generate read counts for each gene in consortium and monospecies conditions, the number of retained read pairs were counted in every sample using HTseq version 0.6.1 for strand‐specific paired‐end reads using intersection_nonempty as overlap resolution mode (Anders, Pyl, & Huber, 2015).

Differential expression of genes between consortium and monospecies conditions was obtained using the DEseq2 package in R (Love, Huber, & Anders, 2014). The independent filtering setting was used in order to increase experiment‐wide power and the Benjamini–Hochberg correction was used to correct for multiple hypothesis testing.

To check if sequencing depth was sufficient, we adapted a method used in biodiversity sampling studies (Colwell et al., 2012), by plotting the proportion of identified coding sequences (CDS) of the WDL1 or WDL7 genome that were expressed (read pair count ≥1) as a function of the sampling size in consortium and monoculture RNAseq libraries. The sampling size was expressed as the number of read pairs that uniquely mapped to CDS of WDL1 or WDL7 in each library. Genes were called differentially expressed between consortium and monoculture conditions when |log 2 fold change| ≥1. We did not take into account the *p*‐values of the differential expression analysis as this would be too restrictive. This was shown before to be a valid approach to analyze differential transcriptomic data (Beliaev et al., 2014; Horinouchi et al., 2010; Suzuki, Horinouchi, & Furusawa, 2014). While this approach is more prone to false positives, this is offset by the subsequent pathway analysis which is more robust to false positives.

### 2.5 Differential gene expression analysis

Analysis of differentially expressed genes was based on the Kyoto Encyclopedia of Genes and Genomes (KEGG) orthology classification of proteins (http://www.genome.jp/kegg/). KEGG identifiers for the CDS of the three genomes of the consortium members were obtained by exporting the amino acid sequences of all CDS from the RAST server and uploading them on the KEGG automatic annotation server (http://www.genome.jp/tools/kaas/). To unveil pathways and other cellular systems underlying the differentially expressed genes between consortium conditions and isolated conditions for both WDL1 and WDL7, the network‐based algorithm PheNetic (De Maeyer, Weytjens, Renkens, De Raedt, & Marchal, 2015) and the Search Brite tool for functional classification using KEGG orthology (Kanehisa, Goto, Sato, Furumichi, & Tanabe, 2012) were used. PheNetic searches for common pathways between differentially expressed genes based on an interaction network. The used interaction networks in WDL1 and WDL7 consisted of metabolic, (de)methylation, and (de)phosphorylation interactions from KEGG (Kanehisa et al., 2012) version 80.0. For WDL1 and WDL7, respectively, interactions documented in any of four *Variovorax* strains (*V. paradoxus* S110, *V. paradoxus* EPS, *V. paradoxus* B4, and *Variovorax* sp. PAMC 2877) and any of two *C. testosteroni* strains (CNB‐2, TK102) were used. The standard parameters were applied, run mode was set to “downstream” and the cost was set to 0.15 for WDL1 and 0.1 for WDL7. These costs were determined by running a sweep over the cost parameter. In order to avoid selecting noise, identified pathways consisting of at most three genes were discarded The subsystem tool from RAST (Aziz et al., 2008) was used to look for additional genes with no KEGG identifier that could be linked with the differentially expressed pathways and other cellular systems. The RNAseq‐derived expression of four genes of WDL1 (*hylA*,* dcaQ*,* catA*, and *phoA*) and five genes of WDL7 (*pcaF*,* glxR*,* pilM*,* pilY1*, and *yrbC*) were validated by real‐time quantitative PCR (qRT‐PCR). For detailed information, see Data S1.

### 2.6 Verification of differential transcription using transcriptional gene fusions

Transcriptional gene fusions between the promotor regions of the glycerate biosynthesis operon (*gcl*) and of the 3‐oxoadipate catabolic operon (*pca*) of WDL7 with the promoterless *gfpmut3.1* in the broad host range vector pRU1097 were constructed in WDL7 and tested for expression in WDL7 monoculture and WDL1/WDL7/WDL6 consortium biofilms as reported in Data S1.

### 2.7 Nucleotide sequence accession numbers

The draft genome sequences of *Variovorax sp*. WDL1, *C. testosteroni* WDL7, and *H. sulfonivorans* WDL6 have been deposited at DDBJ/EMBL/GenBank under the accession numbers LMTS00000000, LMXT00000000, and LMTR00000000, respectively.

## 3 RESULTS

### 3.1 Linuron and 3,4‐DCA degradation performance of consortium and monoculture biofilms

**Figure 1:**
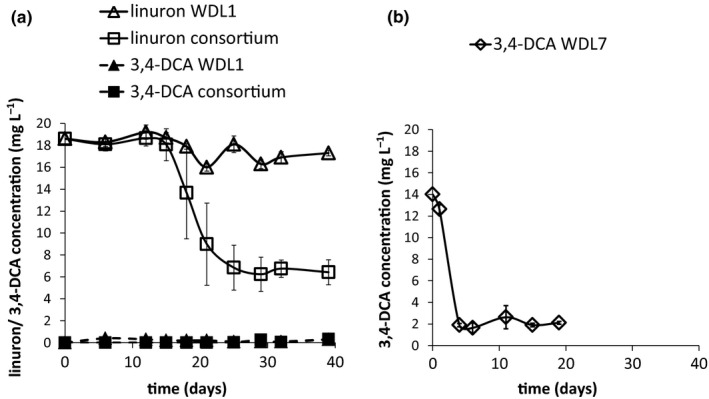
(a) Time lapse effluent concentrations of linuron (solid lines) and 3,4‐dichloroaniline (3,4‐DCA; dotted lines) in flow channels containing WDL1/WDL7/WDL6 consortium (squares) and WDL1 monoculture biofilms (triangles). (b) Time lapse effluent concentration of 3,4‐DCA in flow channels containing WDL7 monoculture biofilms (diamonds). Each data point with error bar represents the mean and standard deviations of three replicate systems

For differential gene expression analysis, biofilms of the consortium containing WDL1, WDL6, and WDL7, as well as monoculture biofilms of WDL1 and WDL7 were grown on MMO supplemented with linuron or 3,4‐DCA as the sole carbon source. The synergistic degradation of linuron by the consortium was evident from the linuron degradation efficiency of consortium biofilms compared to this of WDL1 monoculture biofilms (Figure 1a). The latter degraded linuron inefficiently and effluent concentrations never dropped below 96% of the linuron influent concentration. In the consortium biofilms, the linuron effluent concentration started to decrease after 2 weeks of operation. After 29 days, steady‐state conditions were attained with linuron effluent concentration stagnating around 35% of the influent concentration. Minor accumulation of 3,4‐DCA was observed in both consortium and WDL1 monoculture biofilms, never exceeding 3% and 2% (molar equivalent) of the linuron influent concentration, respectively. In WDL7 monoculture biofilms fed with 3,4‐DCA, effluent 3,4‐DCA concentrations started to decrease after 2 days of operation. After 4 days, steady‐state degradation was obtained and effluent 3,4‐DCA concentrations remained around 14% of the influent concentration (Figure 1b).

### 3.2 Overall analysis and validation of RNAseq data

**Figure 2:**
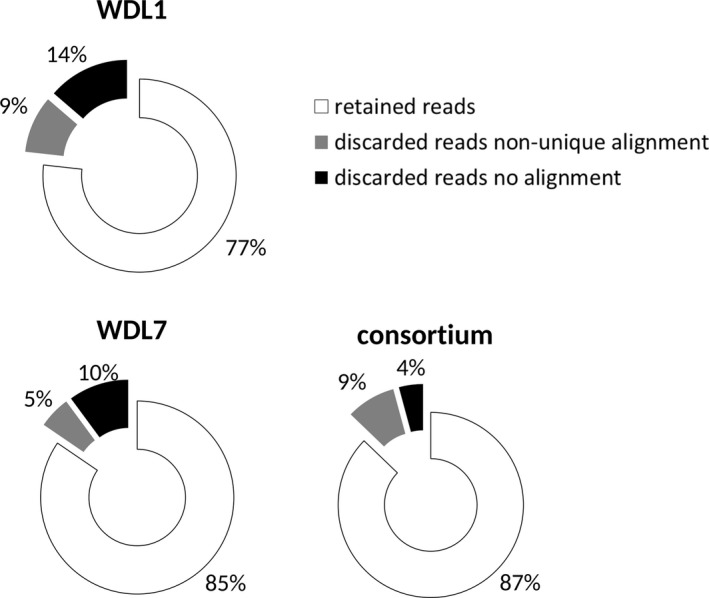
Average percentages of retained and discarded RNAseq read pairs obtained with one WDL1 monoculture (WDL1), three WDL7 monoculture (WDL7), and three consortium (consortium) biofilm samples after mapping the read pairs to the compiled triple‐species reference genome of *Variovorax* sp. WDL1, *Comamonas testosteroni* WDL7, and *Hyphomicrobium sulfonivorans* WDL6

**Figure 3:**
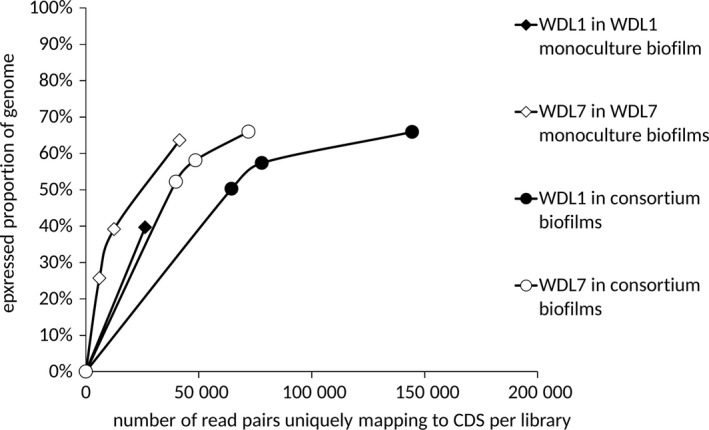
Rarefaction curves showing the proportion of coding sequences (CDSs) of the genome of WDL1 or WDL7 that were expressed (with read pair count ≥1) in the monoculture and consortium biofilms (as determined by RNAseq) as a function of the number of read pairs uniquely mapping to CDSs of WDL1 or WDL7: expressed proportion of WDL1 genome in the WDL1 monoculture biofilms (black diamond), expressed proportion of WDL1 genome in the WDL1/WDL6/WDL7 consortium biofilms (black circles), expressed proportion of WDL7 genome in the WDL7 monoculture biofilms (white diamonds), and expressed proportion of WDL7 genome in the WDL1/WDL6/WDL7 consortium biofilms (white circles)

Samples for transcriptome analysis were taken after 2 weeks of steady linuron or 3,4‐DCA degradation. Sequencing of all cDNA libraries resulted in a total of 74 million read pairs (Table 1). Monoculture and consortium libraries had been multiplexed in a 1:5 concentration ratio for sequencing which was reflected by the numbers of reads from consortium libraries being higher than those from monoculture libraries. However, the number of read pairs between replicates was highly variable (Table 1). After the trimming step, read pairs were mapped on the compiled genome sequences of strains WDL1, WDL6, and WDL7. A challenge for performing RNAseq analysis using transcript data from mixed cultures is the risk that reads will be mapped erroneously to orthologous genes present in the other strains. Therefore, reads that show nonunique alignment on the compiled triple‐species reference genome sequence were discarded. Results show that on average only 9 (±1)% of the reads of the consortium samples were as such discarded. Finally, 76%–88% of the read pairs in the samples could be unambiguously mapped on the compiled triple‐species reference genome sequence (Figure 2). Despite the rRNA depletion treatment, on average 88% of these retained read pairs aligned with rRNA genes. The percentage of retained read pairs that mapped to CDSs was low for all samples: 0.4% for the WDL1 monoculture biofilm, 1.1%–1.5% for the three WDL7 monoculture biofilms, and 0.7%–1.0% for the three consortium biofilms. The number of read pairs mapping to CDSs ranged from 6,008 to 227,025 which is lower than that observed in comparable studies (300,000–1,000,000 read pairs) (Frias‐Lopez & Duran‐Pinedo, 2012; Rosenthal et al., 2011). Rarefaction curve analysis (Figure 3) showed that despite the low number of mapped read pairs, the number of expressed CDS detected in the consortium samples and one WDL7 monoculture sample (50%–66%) was close to the estimated asymptotic value of 70%. On the other hand, the sequencing depth for two WDL7 monoculture replicates and the WDL1 monoculture sample only covered the expression of 26%–40% of the genes, indicating that for those samples a part of the genes with low expression levels remained undetected. Overall, gene expression values obtained for biological replicates of the different samples were highly correlated, with a Pearson's correlation (*r*) of on average 0.99 between pairs of WDL1 in consortium samples, of 0.93 for pairs of WDL7 in consortium samples, and of 0.97 between the WDL7 monoculture samples. RNAseq inferred differential expression levels observed between consortium and monoculture conditions were reassessed by qRT‐PCR for four genes of WDL1 (*hylA*,* dcaQ*,* ccdC*, and *phoA*) and five genes of WDL7 (*pcaF*,* glxR*,* pilM*,* pilY1*, and *yrbC*). Those genes were selected as they were considered as potentially involved in interspecies interactions based on annotation (*hylA*,* dcaQ*,* ccdC*,* pcaF*, and *pilY1*) and/or because their RNAseq‐inferred differential expression levels ranged from underexpression (*pcaF*) over nondifferential (*dcaQ*,* ccdC*,* phoA*,* pilY1*, and *yrbC*) to overexpression (*hylA*,* glxR*, and *pilM*) in consortium conditions. qRT‐PCR‐ and RNAseq‐based values showed a high Pearson's correlation (*r* = 0.97) (Figure S1). The high reproducibility between similar samples and the confirmation of RNAseq‐based differential expression by means of qPCR indicates that one WDL1 monoculture sample is sufficient for correctly analyzing the differential response of WDL1 to consortium growth.

**Table 1:** Summary of sequencing results and read alignment of the RNAseq libraries analyzed in this study

	Replicate	No. of read pairs	No. of filtered read pairs (PHRED score >30)	No. of uniquely mapped read pairs	No. of read pairs uniquely mapped to rRNA	No. of read pairs uniquely mapped to CDS
WDL1	1	8,090,000	7,921,052	6,491,448	6,032,872	26,258
WDL7	1	1,360,000	1,329,361	1,169,256	1,077,552	12,466
	2	470,000	463,034	420,385	384,077	6,008
	3	3,330,000	3,248,923	2,783,049	2,478,562	41,470
Consortium	1	15,850,000	15,441,928	13,920,873	13,080,821	107,069
	2	25,040,000	24,490,945	22,529,095	14,805,214	227,025
	3	20,590,000	20,005,138	18,442,744	17,227,266	134,644
Total		74,720,000	72,900,381	65,756,850	55,086,363	554,940

CDS, coding sequences.

### 3.3 Transcriptional responses in *Variovorax* sp. WDL1 when grown in consortium conditions

**Figure 4:**
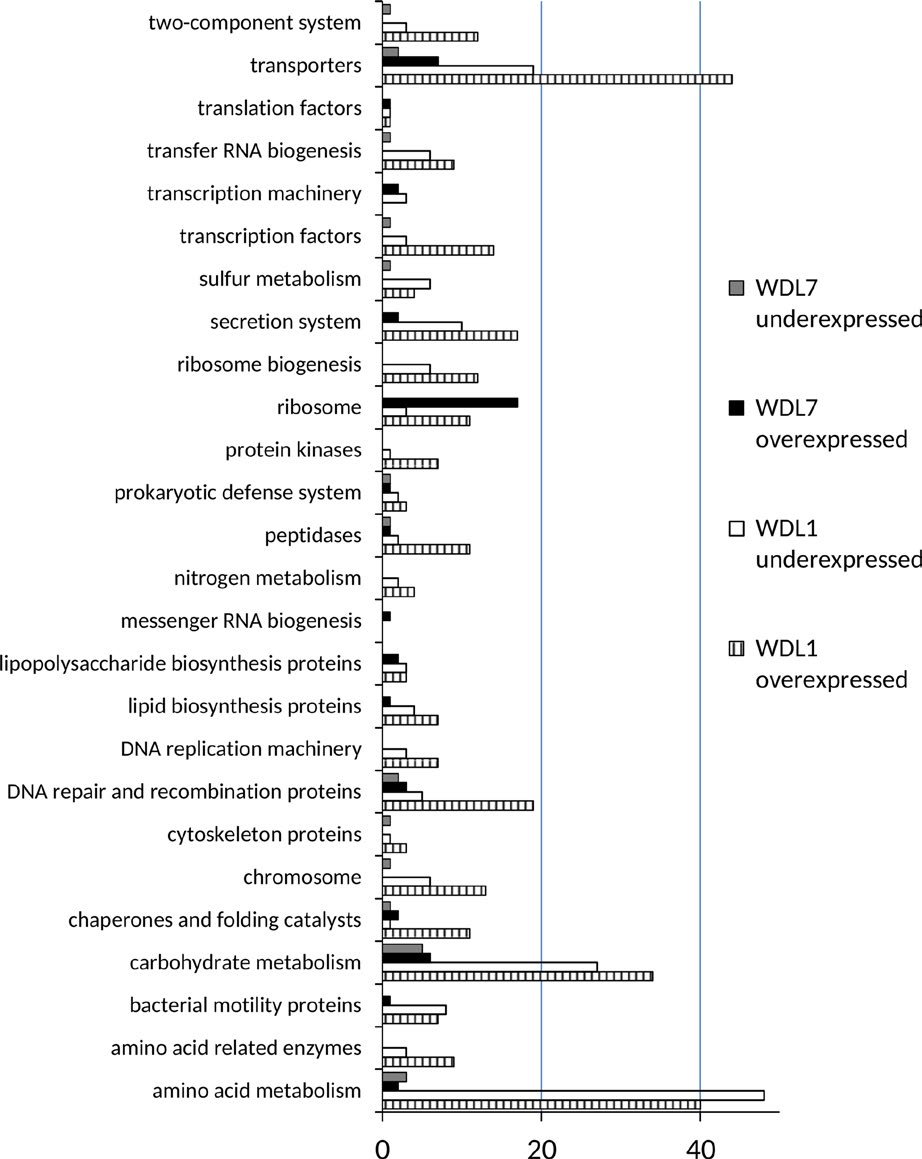
Kyoto Encyclopedia of Genes and Genomes orthology‐based functional categorization of coding sequences that are over‐ or underexpressed in WDL1 and WDL7 when grown in WDL1/WDL6/WDL7 triple species biofilms compared to their growth in monoculture biofilms

**Figure 5:**
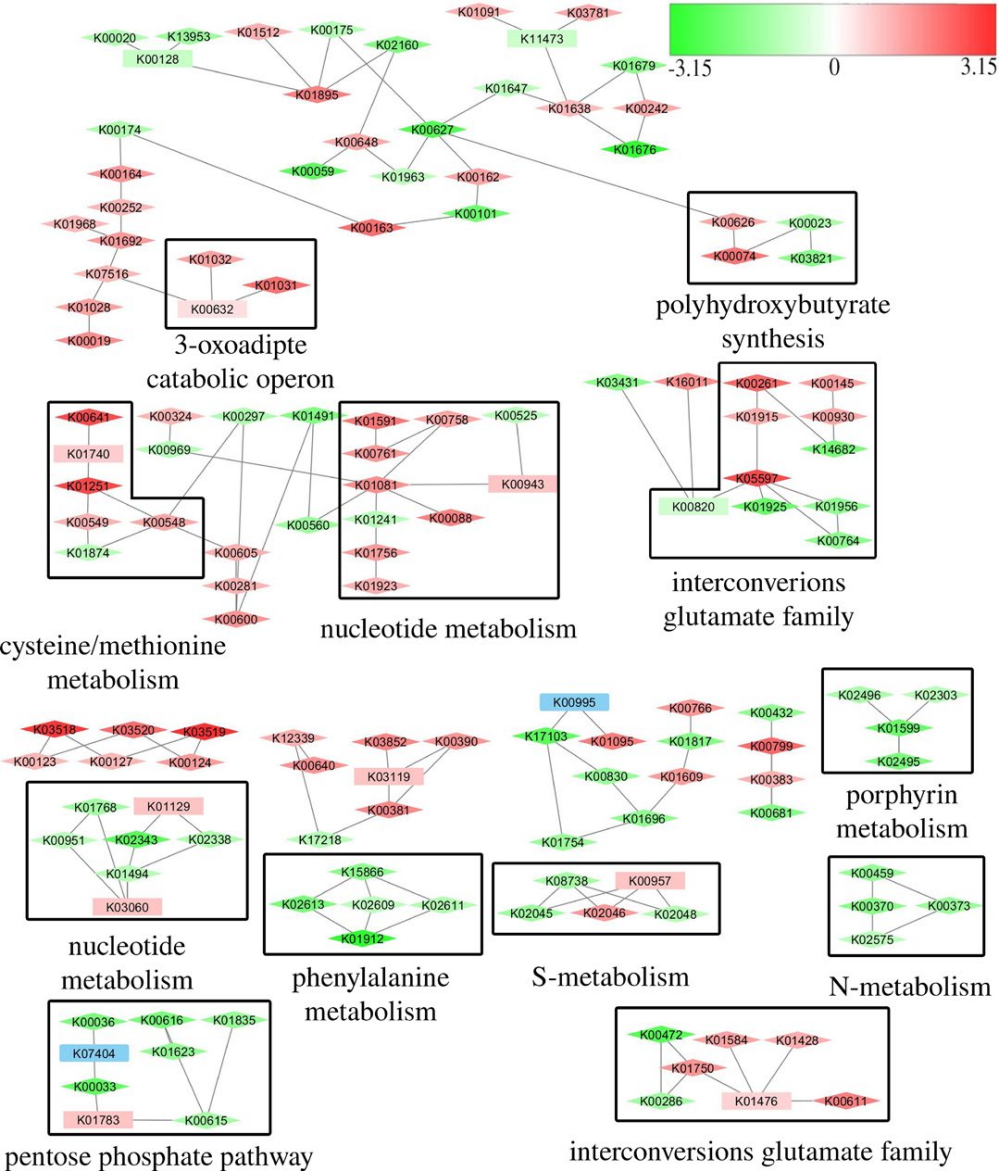
Molecular pathways differentially expressed between consortium and monoculture conditions for WDL1 as inferred by PheNetic. Log2‐fold change in expression is indicated on a color scale from green to red, with red signifying underexpression in consortium conditions and green representing overexpression in consortium conditions. Blue‐highlighted genes are genes for which no transcripts were recorded. Genes that are differentially expressed (|Log2‐fold change| ≥ 1) are indicated by diamond shapes. Discussed pathways are boxed and annotated

In WDL1, 1372 CDSs showed differential expression between monoculture and consortium conditions. The differentially expressed CDSs were functionally categorized based on KEGG orthology (Figure 4) and PheNetic was used to find pathways underlying the differentially expressed genes (Figure 5). Only a few genes of catabolic clusters involved in linuron degradation in WDL1 were differentially expressed. The linuron hydrolase gene *hylA* in WDL1 appeared more than 100‐fold overexpressed in consortium conditions (Table S1), while all three genes of the 3‐oxoadipate catabolic operon (*pcaFIJ*) required for converting the linuron metabolite 3‐oxoadipate into TCA cycle intermediates (K01031, K00632, and K01032), were two‐ to fivefold underexpressed (Figure 5). All other genes putatively involved in linuron degradation to TCA intermediates, that is, all genes belonging to the *dcaQTA1A2B* gene cluster encoding the 3,4‐DCA multicomponent dioxygenase and the *ccdCFDE* gene cluster encoding conversion of chlorocatechols to 3‐oxoadipate were not differentially expressed between consortium and monoculture conditions. The *dcaQTA1A2B*,* ccd*, and *pca* clusters represented about 11%, 5%, and 0.2%, respectively, of the total number of transcript read pairs mapping with CDSs in both consortium conditions and monoculture conditions indicating their high expression in WDL1 regardless of the strain was grown alone or together with WDL7 and WDL6 (Table S2).

The results further showed that overall, general cell metabolism was altered in WDL1 in consortium conditions compared to monoculture conditions. When grown in consortium conditions, 351 enzyme‐encoding genes were differentially expressed genes in WDL1. About 17% of these genes (61 CDS) were involved in carbohydrate metabolism (Figure 4) and several carbohydrate metabolizing pathways were selected by PheNetic (Figure 5). Genes involved in polyhydroxybutyrate synthesis from acetoacetyl‐CoA (K03821, K00626, and K00023) were overexpressed in consortium conditions as well as a large fraction of the genes determining the anabolic pentose phosphate pathway (K00615, K01623, K00616, K01835, K00036, and K00033) including genes encoding the conversion of β‐d‐glucose‐6P to d‐ribulose‐5P and of d‐glyceraldehyde‐3P to d‐ribulose‐5P and/or d‐ribose‐5P (Figure S2). Another important fraction (88 CDS) of the enzyme‐encoding genes that were differentially expressed were involved in amino acid metabolism, and PheNetic selected several differentially expressed pathways that relate to amino acid metabolism. Genes encoding interconversion reactions between molecules of the glutamate family were differentially expressed (Table S1 and Figure S3). Similarly, genes involved in the degradation of phenylacetate to acetyl‐CoA in the phenylalanine metabolism were largely overexpressed in consortium conditions (Figure S4). Moreover, in cysteine and methionine metabolism, several genes that were involved in cycling of the central metabolite *S*‐adenosylmethionine (K01251, K00548, K00549, K00789) and genes involved in cysteine synthesis (K12339, K00640) were underexpressed in consortium conditions (Figure S5). The differential expression of several genes encoding transporters involved in the uptake of amino acid and carbohydrate molecules was also indicative of a metabolic response upon coculturing. Most of the transporters are members of the ATP‐binding cassette superfamily (Table S1). Changes in general cell metabolism were further suggested by altered expression in pathways involved in sulfur and nitrogen transport and metabolism. Pathways involved in sulfur transport and metabolism were underexpressed in consortium conditions such as the synthesis of the sulfur‐containing metabolite thiamine (K03147, K03154) as well as an operon spanning three genes (K00390, K00381, K00957) involved in assimilatory sulfate reduction. Differentially expressed genes associated with nitrogen metabolism (Table S1) included genes involved in ammonium and nitrate/nitrite transport (K03320, K02575), assimilation of inorganic nitrogen via glutamine synthetase (K00370, K00373, K01915), and several nitrogen regulatory and sensor proteins (K07673, K07712, K07708).

Also, DNA metabolism appeared to be affected. Increased DNA synthesis by WDL1 in consortium conditions is suggested by the underexpression of degradation of purine and pyrimidine nucleotides (K00758, K01081) and overexpression of formation of deoxynucleotides (K02343, K02338, K01494, K00525) (Figures S6 and S7). In accordance with the suggested increased DNA synthesis of WDL1, 20 genes involved in different mechanisms of DNA repair and recombination were overexpressed (Table S1). Also, a part of the porphyrin metabolism was selected by PheNetic. Additional manual analysis showed that genes involved in heme production (K01599, K02495, K02492, and K01698) were overexpressed in consortium conditions (Figure S8).

Besides the metabolic pathways that were mainly identified by PheNetic, additional cellular systems were found to be differentially expressed between growth conditions in WDL1 (Figure 4). Several of those systems are related to cell to cell interactions. These included overexpression under consortium conditions of eight of thirteen genes coding for a Type VI secretion system (T6SS), two genes that encode a toxin/antitoxin pair participating in systems mediating contact‐dependent inhibition (CDI), a gene encoding a putative adhesin, and genes encoding a putative quorum‐sensing circuit (Table S1). Interestingly, the latter are located just downstream of linuron hydrolase encoding gene *hylA* and encode a LuxR‐type transcriptional regulator (K18098) and LuxI‐type acyl‐homoserine lactone synthase (K18096).

Another set of genes with altered expression are associated with stress response indicating that WDL1 experiences cellular stress in consortium conditions. *rpoH* and *hrcA* were overexpressed in consortium compared to monoculture conditions. *rpoH* encodes sigma factor σ^32^ which controls genes involved in heat shock response and protein homeostasis. Genes encoding chaperones DnaK, DnaJ, GroEL, GroES, HtpG, and ClpB, and a zinc protease were all overexpressed, whereas *grpE* was underexpressed (Table S1).

Other responses of WDL1 in consortium conditions included functions that could not directly be related to metabolism, stress, or cell to cell interactions. Eleven genes involved in copper homeostasis were overexpressed in consortium conditions, including a copper‐responsive two‐component system controlling copper efflux (Table S1). Also, several genes of the central control region of an IncP1 plasmid encoding regulatory and stability functions (Krol et al., 2012) were overexpressed in consortium conditions (Table S1).

### 3.4 Transcriptional responses elicited in *C. testosteroni* WDL7 when grown in consortium biofilms

Only 169 CDSs in WDL7 showed differential expression when comparing monoculture with consortium conditions. Similar to WDL1, only a few genes of the gene clusters predicted to be involved in 3,4‐DCA degradation were differentially expressed in WDL7. The *dcaB* gene of the *dcaQTA1A2B* cluster encoding the oxygenation of 3,4‐DCA in WDL7 was twofold underexpressed in consortium conditions (Table S1). Furthermore, in contrast to WDL1, only *pcaF* of the *pcaFIJ* operon, that is, the gene encoding acetyl‐CoA acetyltransferase (APV28_0875; Table S1), was threefold underexpressed in consortium conditions. However, the latter was not be confirmed by transcriptional fusion reporter analysis (Figure S9a). *catAB*,* CMBL*, and *tfdF* encode conversion of chlorocatechol to 3‐oxoadipate in WDL7 (Wu, Mohanty, Chia, & Cao, 2016), and were not differentially expressed between consortium and monoculture conditions. As in WDL1, most 3,4‐DCA catabolic genes (*dcaQTA1A2*,* catAB*,* CMBL*,* tfdF*, and *pcaIJ*) were not differentially expressed and all 3,4‐DCA catabolic genes represented together more than 25% of the transcript reads indicating a high expression of the 3,4‐DCA catabolic pathway in WDL7 (Table S2).

**Figure 6:**
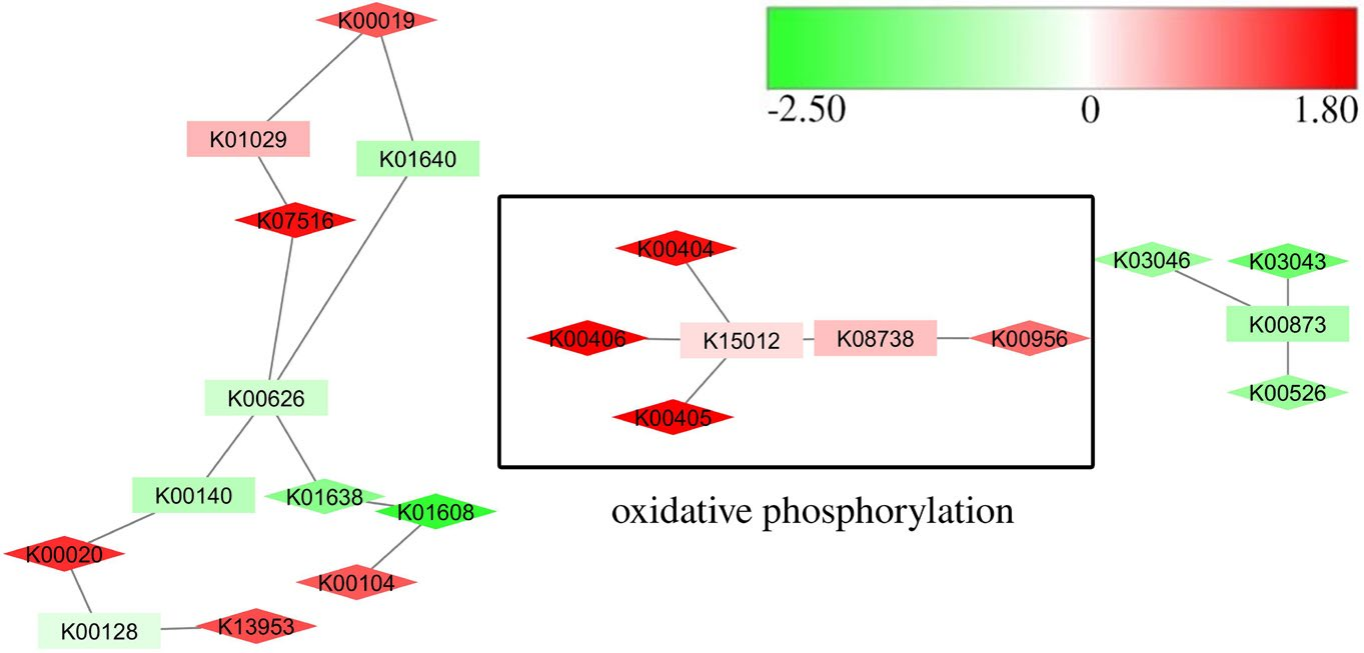
Molecular pathways differentially expressed between consortium and monoculture conditions for WDL7 as inferred by PheNetic. Log2‐fold change in expression is indicated on a color scale from green to red, with red signifying underexpression in consortium conditions and green representing overexpression in consortium conditions. Genes that are differentially expressed (|Log2‐fold change| ≥ 1) are indicated by diamond shapes. Discussed pathways are boxed and annotated

One system that was selected by PheNetic as clearly underexpressed in WDL7 under consortium conditions was the high‐affinity *cbb3*‐type cytochrome *c* oxidase (K00404, K00405, K00406; Figure 6). None of the other three terminal respiratory oxidase gene clusters in WDL7 showed altered expression in consortium conditions compared to monoculture conditions. Another operon that was strongly affected in WDL7 in consortium conditions was the glycerate biosynthesis operon (*gcl*; K01608, K00042, K01816). This operon is involved in the conversion of glyoxylate to glycerate and showed clear overexpression in consortium versus monoculture conditions (Table S1). Its overexpression in consortium conditions was confirmed by transcriptional fusion reporter analysis (Figure S9b). Furthermore, 17 of 53 genes coding for ribosomal proteins in WDL7 were overexpressed in consortium conditions. Nearly one third of the genes belonging to an unknown prophage element were underexpressed (Table S1). In contrast to WDL1, no differentially expressed genes involved in cell to cell interaction were identified in WDL7, while overexpression was observed for only one gene that is related to stress (APV28_0402) and one gene related to DNA repair and recombination (APV28_2634).

## 4 DISCUSSION

### 4.1 Expression of linuron catabolic pathway genes in WDL1 changes upon coculturing of the consortium members

The WDL1 *hylA* gene encoding linuron hydrolase showed strong overexpression in consortium conditions. The higher expression of *hylA* per cell in WDL1 under consortium conditions is remarkable but can be at least partially be explained by recent observations that (1) WDL1 consists of two subpopulations, one carrying *hylA* and lacking the 3,4‐DCA multicomponent dioxygenase gene cluster and one that carries only the 3,4‐DCA pathway, and (2) the linuron‐hydrolyzing WDL1 subpopulation becomes more abundant (up to 10‐fold) when grown in consortium conditions than when grown in monoculture, as shown by qPCR targeting *hylA* and the *dca* gene cluster in other identically operated biofilm experiments (P. Albers, unpubl. results). Regardless of the dynamics of the linuron‐hydrolyzing WDL1 subpopulation, the overall higher abundance of *hylA* transcripts in consortium conditions suggests an increased production of HylA, which can obviously be linked with the enhanced degradation of linuron in consortium biofilms. The *pcaFIJ* operon consisting of three genes encoding the last steps in linuron conversion to TCA cycle intermediates was underexpressed in WDL1 in consortium conditions. As in *Sinorhizobium meliloti*, the *pca* operon of WDL1 is preceded by a regulatory gene that encodes an IclR‐type regulator which upon interaction with 3‐oxoadipate results in induction of the sinorhizobial *pca* operon (MacLean, MacPherson, Aneja, & Finan, 2006). This suggests that the *pca* operon in WDL1 is also inducible by 3‐oxoadipate and that underexpression is possibly due to less 3‐oxoadipate being used by WDL1 cells during consortium growth. On the other hand, it could be speculated that a lower expression of the *pca* operon in WDL1 is a consequence of catabolic repression by metabolic waste products or nutrients supplied by the other strains (Bleichrodt, Fischer, & Gerischer, 2010). Regarding the linuron catabolic genes in WDL7, *dcaB* was the only gene of the *dca* operon of WDL7 that was slightly underexpressed in consortium condition. Other genes of the linuron catabolic operons of WDL7 were all highly but not differentially expressed, supporting the assumption that 3,4‐DCA is a major carbon source for WDL7, not alone in monoculture, but also in consortium biofilm conditions (Dejonghe et al., 2003). This observation further confirms that there is metabolic association between WDL1 and WDL7 during linuron degradation.

### 4.2 Coculturing of strains modulates additional metabolic pathways in the linuron‐degrading consortium

As reported above, we recently observed that WDL1 consists of a linuron‐hydrolyzing and a 3,4‐DCA‐oxidizing subpopulation. Considering that the linuron‐hydrolyzing WDL1 subpopulation retrieves no energy or carbon from linuron hydrolysis, its growth on linuron in consortium conditions can only be explained by the uptake and metabolism of alternative carbon sources produced by the other consortium members during linuron degradation. Cross‐feeding and growth on compounds other than linuron metabolites is indeed supported by the many differentially expressed genes, particularly in WDL1, that can be linked to the exchange of metabolites with their environment. First, the high number of genes coding for functions involved in transport and two‐component systems in WDL1 that are overexpressed in consortium conditions, likely reflects a substantial change in the chemical composition of the local environment surrounding WDL1 cells. Since the three consortium members are closely associated when grown together as a biofilm (Breugelmans et al., 2008), each strain is confronted with the metabolic footprint of the other strains. The metabolic footprint, that is, the ensemble of metabolites in the extracellular space as a result of uptake of nutrients and the excretion of metabolites, was previously shown to be, among other factors, a species‐specific trait (Kell et al., 2005; Palama et al., 2016). A similar change in expression of genes coding for membrane proteins in multispecies biofilms of soil bacteria was observed before (Hansen, Ren, Burmolle, & Sorensen, 2017). Second, the altered expression of genes encoding enzymes involved in metabolic pathways is similarly indicative of cross‐feeding between the consortium members. Amino acids appear as one such type of molecules exchanged in the consortium as suggested by the altered expression in WDL1 of genes involved in amino acid metabolism and transport, but also by a change in expression of genes involved in nitrogen regulation, uptake of inorganic nitrogen, and assimilation via glutamine synthetase. Differential expression of genes involved in nitrogen metabolism in bacteria as a response to multiculture growth was previously observed and suggested to be associated with the exchange of amino acids or other nitrogen‐containing compounds between consortium partners (Beliaev et al., 2014; Cerqueda‐Garcia, Martinez‐Castilla, Falcon, & Delaye, 2014; Tai et al., 2009; Wenter et al., 2010). Interestingly, addition of amino acids was previously shown to enhance degradation of linuron in monocultures of linuron‐degrading *Variovorax* strains that were recovered from linuron‐degrading consortia with compositions similar to the tripartite WDL1/WDL6/WDL7 consortium (Breugelmans et al., 2007). Alternatively, the altered nitrogen metabolism in WDL1 can be merely a consequence of the loss of 3,4‐DCA as the nitrogen‐delivering metabolite of linuron degradation by the WDL1 cells in consortium conditions due to the more efficient uptake by WDL7. The underexpression of genes involved in the biosynthesis of sulfur‐containing compounds such as thiamine, methionine, and cysteine in consortium conditions in WDL1 could point toward a lower dependence on de novo synthesis of these compounds when they are obtained from other strains in consortium conditions. This was also observed previously in a cyanobacterial‐heterotrophic coculture (Beliaev et al., 2014).

Compared to WDL1, WDL7 shows a less extensive metabolic response upon coculturing. Only a clear overexpression of the glycerate biosynthesis *gcl* operon was observed in WDL7 during consortium growth. This operon was shown to be induced by glyoxylate in *Escherichia coli* (Teramoto et al., 2010), suggesting that WDL7 senses a higher concentration of glyoxylate when grown in consortium conditions and thus that glyoxylate is a candidate metabolite that WDL7 could receive during consortium growth. As WDL7 does not show a strong change in regulation of metabolic pathways between growth conditions, 3,4‐DCA seems to remain the main carbon and energy source of WDL7 both in monoculture and consortium biofilm conditions. In contrast to the nondifferential expression of metabolic pathways in WDL7, its growth rate does seem to increase in consortium conditions based on the abundant overexpression of one third of WDL7 genes encoding for ribosomal proteins. This has been described before in other consortia and was suggested to be a metric of in situ growth rate (Gonzalez‐Torres et al., 2015; Perez et al., 2015). A higher growth rate is also expected for WDL1 in consortium conditions from the increase of DNA synthesis (Ward & Glaser, 1971).

### 4.3 Coculturing of consortium strains triggers a stress response in WDL1

Growth in consortium conditions engenders the overexpression of stress‐related proteins in WDL1, like the heat shock regulon. Some of these molecular chaperones were also found to be overexpressed in a phototrophic bacterial consortium (Wenter et al., 2010) and in a halophilic coculture of closely related strains (Gonzalez‐Torres et al., 2015) indicating that a stress response upon cogrowth of strains mutually benefiting from the interaction is not unique to the linuron‐degrading consortium. Also several genes involved in DNA repair and recombination appear to be upregulated indicating that increased DNA damage occurs in WDL1 under consortium conditions. DNA damage can be caused by change in metabolic activity resulting in an enhanced production of reactive oxygen species that are continuously generated during metabolism (Zgur‐Bertok, 2013), but can also be caused by exogenous agents such as toxins (Dwyer, Kohanski, Hayete, & Collins, 2007). On the other hand, increased DNA repair and recombination might also be due to the increased DNA synthesis in WDL1 in consortium conditions as indicated by the underexpression of degradation of purine and pyrimidine nucleotides and overexpression of formation of deoxynucleotides. Nevertheless, coexistence in the linuron‐degrading consortium appears to be experienced by WDL1 as a stressful situation implying that the ecological interactions between the consortium members are more complex than merely the exchange of metabolites. Nadell, Xavier, and Foster (2009) stated before that the benefits of mutualistic interactions do not exclude the existence of conflicts between tightly associated strains as they might be competing for other limited resources such as oxygen or space.

### 4.4 Potential involvement of cell to cell interactions in shaping the linuron‐degrading consortium

In this study, we observed two types of contact‐dependent interaction systems (T6SS and CDI) that were overexpressed in consortium conditions in WDL1. In both T6SS and CDI, toxic effector proteins and antitoxins form a functional pair and mediate growth inhibition of neighboring competitor cells that do not produce the identical toxin/antitoxin pair (Garcia, Perault, Marlatt, & Cotter, 2016; Russell, Peterson, & Mougous, 2014). As explained above, strains that mutually benefit from coexistence can at the same time be competitors for other resources, and the T6SS and CDI systems might be used by WDL1 for contact‐dependent interference competition with WDL6 and WDL7. However, CDI and T6SS can also mediate cooperation and communication between bacteria expressing the same toxin–antitoxin system in which case they are immune to each other's toxins and are called “self”‐bacteria. Alternative ecological roles beyond competition have been proposed for CDI and T6SS, where the toxin protein could be interpreted as a contact‐dependent signal molecule by “self”‐bacteria. In this way, toxin–antitoxin systems not only can contribute to community architecture, but they can also enforce cooperative behavior in “self”‐bacteria when the toxin–antitoxin pair is coregulated with genes coding for social traits by killing “self”‐bacteria that do not express social traits (Russell et al., 2014). In that context, it was recently shown that the CDI system of *Burkholderia dolosa* alters gene expression in *B. thailandensis* showing that “self”‐bacteria can belong to different species as long as they produce the same toxin–antitoxin pair (Garcia et al., 2016). However, we only identified T6SS or CDI toxin or antitoxin genes in WDL1 and not in the other consortium members. The involvement of interspecies contact‐independent signaling in the consortium is further suggested by the higher transcript abundance of an *luxR/luxI*‐like quorum‐sensing module in WDL1 which is indicative of a higher concentration of autoinducer molecules produced by WDL1 in consortium biofilms. Intriguingly, this quorum‐sensing gene module is only present in the linuron‐hydrolyzing subpopulation of WDL1 where it is located just adjacent to the highly overexpressed linuron hydrolase gene *hylA* which prompts the question if this quorum‐sensing system may control *hylA* expression and the linuron hydrolysis phenotype in WDL1. Autoinducer production is typically enhanced when a bacterial population reaches a critical population density (quorum). However, the increased transcript abundance of the *luxI*‐ and *luxR*‐like genes in WDL1 in consortium conditions is only 3‐ and 15‐fold, respectively, which is not higher than the 10‐fold increased abundance of the linuron‐hydrolyzing WDL1 subpopulation in consortium conditions (P. Albers, unpubl. results). These data suggest that the higher transcript abundance of quorum‐sensing genes in consortium conditions is merely a consequence of an increased abundance of the linuron‐hydrolyzing WDL1 subpopulation instead of being caused by a positive feedback loop mechanism of quorum‐sensing genes upon the linuron‐hydrolyzing WDL1 subpopulation reaching a critical quorum. Also, it is not known whether the quorum‐sensing mechanism controls only intraspecific cellular functions or if it is also triggering cellular responses in the other consortium members. No *luxR*‐ or *luxI*‐like genes were found in WDL6. However, a *luxR*‐type regulatory gene with autoinducer‐binding domain (PF03472.13) is present in WDL7 (APV28_3331), whereas no *luxI‐*like gene was identified suggesting that WDL7 does not produce autoinducers. LuxR‐homologs are commonly found in bacteria that are not producing autoinducer molecules (Subramoni & Venturi, 2009). Some of these so‐called “LuxR‐solos” detect and respond to autoinducer molecules produced by other species, such as the quorum‐sensing LuxR‐type regulator SdiA in *E. coli* and *Salmonella* strains controlling cellular functions such as adhesion, putative Type VI secretion, and virulence and that respond in vivo to autoinducers produced by other species such as *Aeromonas hydrophila* in the gastrointestinal tract of turtles (Sabag‐Daigle, Dyszel, Gonzalez, Ali, & Ahmer, 2015; Subramoni & Venturi, 2009).

## 5 CONCLUSION

Using a differential transcriptomic approach, we revealed that next to metabolic association between the members of a linuron‐degrading consortium, additional cross‐feeding interactions are expected to be present and that amino acids are one type of metabolites exchanged between the consortium members that in particular are used by WDL1 for growth. In comparison to WDL7, WDL1 shows a more extensive response upon coculturing with WDL6 and WDL7, including the increased expression of *hylA* encoding linuron hydrolase which can be directly linked with enhanced linuron degradation and a stress response. Furthermore, several cell to cell interaction systems were overexpressed that could be involved in interspecies signaling such as quorum sensing, CDI, and Type VI secretion. Whether or not those signaling systems contribute to the well functioning of the consortium remains to be elucidated. On the contrary, Type VI secretion and CDI could also be used by WDL1 in interference competition with WDL6 or WDL7. This raises the question if synergistic linuron degradation by the consortium involves true adaptive cooperation or is rather a byproduct of selfish interactions between the consortium strains that are competing for other nutrients and space. The apparent experience of stress by WDL1 favors the latter hypothesis. Furthermore, a large number of differentially expressed genes in WDL1 and WDL7 were coding for hypothetical, putative, and unknown proteins which was also observed in similar studies with other consortia (Frias‐Lopez & Duran‐Pinedo, 2012; Gonzalez‐Torres et al., 2015; Kimes, Lopez‐Perez, Auso, Ghai, & Rodriguez‐Valera, 2014). These uncharacterized proteins might include novel functions that are important for the well‐functioning of consortia and their study is of interest for gaining more insight into the synergistic mechanisms in bacterial consortia.

## CONFLICT OF INTEREST

None declared.

## References

[mbo3559-bib-0001] Anders, S. , Pyl, P. T. , & Huber, W. (2015). HTSeq–a Python framework to work with high‐throughput sequencing data. Bioinformatics, 31, 166–169. 25260700 10.1093/bioinformatics/btu638 PMC4287950

[mbo3559-bib-0002] Aziz, R. K. , Bartels, D. , Best, A. A. , DeJongh, M. , Disz, T. , Edwards, R. A. , … Zagnitko, O. (2008). The RAST Server: Rapid annotations using subsystems technology. BMC Genomics, 9, 75. 18261238 10.1186/1471-2164-9-75 PMC2265698

[mbo3559-bib-0003] Beliaev, A. S. , Romine, M. F. , Serres, M. , Bernstein, H. C. , Linggi, B. E. , Markillie, L. M. , … Konopka, A. (2014). Inference of interactions in cyanobacterial‐heterotrophic co‐cultures via transcriptome sequencing. ISME Journal, 8, 2243–2255. 24781900 10.1038/ismej.2014.69 PMC4992078

[mbo3559-bib-0004] Bers, K. , Batisson, I. , Proost, P. , Wattiez, R. , De Mot, R. , & Springael, D. (2013). HylA, an alternative hydrolase for initiation of catabolism of the phenylurea herbicide linuron in *Variovorax* sp. strains. Applied and Environment Microbiology, 79, 5258–5263. 10.1128/AEM.01478-13 PMC3753977 23811502

[mbo3559-bib-0005] Bleichrodt, F. S. , Fischer, R. , & Gerischer, U. C. (2010). The β‐ketoadipate pathway of *Acinetobacter bayly*i undergoes carbon catabolite repression, cross‐regulation and vertical regulation, and is affected by Crc. Microbiology, 156, 1313–1322. 20110298 10.1099/mic.0.037424-0

[mbo3559-bib-0006] Boon, N. , Goris, J. , De Vos, P. , Verstraete, W. , & Top, E. M. (2001). Genetic diversity among 3‐chloroaniline‐ and aniline‐degrading strains of the *Comamonadaceae* . Applied and Environment Microbiology, 67, 1107–1115. 10.1128/AEM.67.3.1107-1115.2001 PMC92702 11229899

[mbo3559-bib-0007] Breugelmans, P. , Barken, K. B. , Tolker‐Nielsen, T. , Hofkens, J. , Dejonghe, W. , & Springael, D. (2008). Architecture and spatial organization in a triple‐species bacterial biofilm synergistically degrading the phenylurea herbicide linuron. FEMS Microbiology Ecology, 64, 271–282. 18373685 10.1111/j.1574-6941.2008.00470.x

[mbo3559-bib-0008] Breugelmans, P. , D'Huys, P. J. , De Mot, R. , & Springael, D. (2007). Characterization of novel linuron‐mineralizing bacterial consortia enriched from long‐term linuron‐treated agricultural soils. FEMS Microbiology Ecology, 62, 374–385. 17991021 10.1111/j.1574-6941.2007.00391.x

[mbo3559-bib-0009] Cerqueda‐Garcia, D. , Martinez‐Castilla, L. P. , Falcon, L. I. , & Delaye, L. (2014). Metabolic analysis of *Chlorobium chlorochromatii* CaD3 reveals clues of the symbiosis in *Chlorochromatium aggregatum* . ISME Journal, 8, 991–998. 24285361 10.1038/ismej.2013.207 PMC3996686

[mbo3559-bib-0010] Colwell, R. K. , Chao, A. , Gotelli, N. J. , Lin, S.‐Y. , Mao, C. X. , Chazdon, R. L. , & Longino, J. T. (2012). Models and estimators linking individual‐based and sample‐based rarefaction, extrapolation and comparison of assemblages. Journal of Plant Ecology, 5, 3–21.

[mbo3559-bib-0011] De Maeyer, D. , Weytjens, B. , Renkens, J. , De Raedt, L. , & Marchal, K. (2015). PheNetic: Network‐based interpretation of molecular profiling data. Nucleic Acids Research, 43, W244–W250. 25878035 10.1093/nar/gkv347 PMC4489255

[mbo3559-bib-0012] Dealtry, S. , Nour, E. H. , Holmsgaard, P. N. , Ding, G. C. , Weichelt, V. , Dunon, V. , … Smalla, K. (2016). Exploring the complex response to linuron of bacterial communities from biopurification systems by means of cultivation‐independent methods. FEMS Microbiology Ecology, 92, pii: fiv157. 10.1093/femsec/fiv157 26705572

[mbo3559-bib-0013] Dejonghe, W. , Berteloot, E. , Goris, J. , Boon, N. , Crul, K. , Maertens, S. , … Top, E. M. (2003). Synergistic degradation of linuron by a bacterial consortium and isolation of a single linuron‐degrading *Variovorax* strain. Applied and Environment Microbiology, 69, 1532–1541. 10.1128/AEM.69.3.1532-1541.2003 PMC150106 12620840

[mbo3559-bib-0014] Dwyer, D. J. , Kohanski, M. A. , Hayete, B. , & Collins, J. J. (2007). Gyrase inhibitors induce an oxidative damage cellular death pathway in *Escherichia coli* . Molecular Systems Biology, 3, 91. 17353933 10.1038/msb4100135 PMC1847949

[mbo3559-bib-0015] Foster, K. R. , & Bell, T. (2012). Competition, not cooperation, dominates interactions among culturable microbial species. Current Biology, 22, 1845–1850. 22959348 10.1016/j.cub.2012.08.005

[mbo3559-bib-0016] Frias‐Lopez, J. , & Duran‐Pinedo, A. (2012). Effect of periodontal pathogens on the metatranscriptome of a healthy multispecies biofilm model. Journal of Bacteriology, 194, 2082–2095. 22328675 10.1128/JB.06328-11 PMC3318478

[mbo3559-bib-0017] Garbeva, P. , Silby, M. W. , Raaijmakers, J. M. , Levy, S. B. , & Boer, W. (2011). Transcriptional and antagonistic responses of *Pseudomonas fluorescens* Pf0‐1 to phylogenetically different bacterial competitors. ISME Journal, 5, 973–985. 21228890 10.1038/ismej.2010.196 PMC3131853

[mbo3559-bib-0018] Garcia, E. C. , Perault, A. I. , Marlatt, S. A. , & Cotter, P. A. (2016). Interbacterial signaling via *Burkholderia* contact‐dependent growth inhibition system proteins. Proceedings of the National Academy of Sciences of the United States of America, 113, 8296–8301. 27335458 10.1073/pnas.1606323113 PMC4961174

[mbo3559-bib-0019] Gonzalez‐Torres, P. , Pryszcz, L. P. , Santos, F. , Martinez‐Garcia, M. , Gabaldon, T. , & Anton, J. (2015). Interactions between closely related bacterial strains are revealed by deep transcriptome sequencing. Applied and Environment Microbiology, 81, 8445–8456. 10.1128/AEM.02690-15 PMC4644643 26431969

[mbo3559-bib-0501] Hansen, L. B. , Ren, D ., Burmolle, M. , & Sorensen, S. J . (2017). Distinct gene expression profile of *Xanthomonas retroflexus* engaged in synergistic multispecies biofilm formation. ISME Journal, 11, 300–303. 27505346 10.1038/ismej.2016.107 PMC5315472

[mbo3559-bib-0020] Horemans, B. , Albers, P. , & Springael, D. (2016). The biofilm concept from a bioremediation perspective In Lear G. (Ed.), Biofilms in bioremediation (pp. 23–40). Poole, UK: Caister Academic Press.

[mbo3559-bib-0021] Horemans, B. , Bers, K. , Ruiz‐Romero, E. , Pose‐Juan, E. , Dunon, V. , De Mot, R. , & Springael, D. (2016). Functional redundancy of linuron degradation in microbial communities of agricultural soil and biopurification systems. Applied and Environment Microbiology, 82, 2843–2853. 10.1128/AEM.04018-15 PMC4836412 26944844

[mbo3559-bib-0022] Horemans, B. , Hofkens, J. , Smolders, E. , & Springael, D. (2014). Biofilm formation of a bacterial consortium on linuron at micropollutant concentrations in continuous flow chambers and the impact of dissolved organic matter. FEMS Microbiology Ecology, 88, 184–194. 24410802 10.1111/1574-6941.12280

[mbo3559-bib-0023] Horemans, B. , Smolders, E. , & Springael, D. (2013). Carbon source utilization profiles suggest additional metabolic interactions in a synergistic linuron‐degrading bacterial consortium. FEMS Microbiology Ecology, 84, 24–34. 23078547 10.1111/1574-6941.12033

[mbo3559-bib-0024] Horinouchi, T. , Tamaoka, K. , Furusawa, C. , Ono, N. , Suzuki, S. , Hirasawa, T. , … Shimizu, H. (2010). Transcriptome analysis of parallel‐evolved *Escherichia coli* strains under ethanol stress. BMC Genomics, 11, 579. 20955615 10.1186/1471-2164-11-579 PMC3091726

[mbo3559-bib-0025] Ilut, D. C. , Coate, J. E. , Luciano, A. K. , Owens, T. G. , May, G. D. , Farmer, A. , & Doyle, J. J. (2012). A comparative transcriptomic study of an allotetraploid and its diploid progenitors illustrates the unique advantages and challenges of RNA‐seq in plant species. American Journal of Botany, 99, 383–396. 22301896 10.3732/ajb.1100312

[mbo3559-bib-0026] Kanehisa, M. , Goto, S. , Sato, Y. , Furumichi, M. , & Tanabe, M. (2012). KEGG for integration and interpretation of large‐scale molecular data sets. Nucleic Acids Research, 40, D109–D114. 22080510 10.1093/nar/gkr988 PMC3245020

[mbo3559-bib-0028] Kell, D. B. , Brown, M. , Davey, H. M. , Dunn, W. B. , Spasic, I. , & Oliver, S. G. (2005). Metabolic footprinting and systems biology: The medium is the message. Nature Reviews Microbiology, 3, 557–565. 15953932 10.1038/nrmicro1177

[mbo3559-bib-0029] Kimes, N. E. , Lopez‐Perez, M. , Auso, E. , Ghai, R. , & Rodriguez‐Valera, F. (2014). RNA sequencing provides evidence for functional variability between naturally co‐existing *Alteromonas macleodii* lineages. BMC Genomics, 15, 938. 25344729 10.1186/1471-2164-15-938 PMC4223743

[mbo3559-bib-0030] Krol, J. E. , Penrod, J. T. , McCaslin, H. , Rogers, L. M. , Yano, H. , Stancik, A. D. , … Top, E. M. (2012). Role of IncP‐1β plasmids pWDL7:rfp and pNB8c in chloroaniline catabolism as determined by genomic and functional analyses. Applied and Environment Microbiology, 78, 828–838. 10.1128/AEM.07480-11 PMC3264110 22101050

[mbo3559-bib-0031] Love, M. I. , Huber, W. , & Anders, S. (2014). Moderated estimation of fold change and dispersion for RNA‐seq data with DESeq2. Genome Biology, 15, 550. 25516281 10.1186/s13059-014-0550-8 PMC4302049

[mbo3559-bib-0032] MacLean, A. M. , MacPherson, G. , Aneja, P. , & Finan, T. M. (2006). Characterization of the β‐ketoadipate pathway in *Sinorhizobium meliloti* . Applied and Environment Microbiology, 72, 5403–5413. 10.1128/AEM.00580-06 PMC1538742 16885292

[mbo3559-bib-0033] Nadell, C. D. , Xavier, J. B. , & Foster, K. R. (2009). The sociobiology of biofilms. FEMS Microbiology Reviews, 33, 206–224. 19067751 10.1111/j.1574-6976.2008.00150.x

[mbo3559-bib-0034] Palama, T. L. , Canard, I. , Rautureau, G. J. , Mirande, C. , Chatellier, S. , & Elena‐Herrmann, B. (2016). Identification of bacterial species by untargeted NMR spectroscopy of the exo‐metabolome. Analyst, 141, 4558–4561. 27349704 10.1039/c6an00393a

[mbo3559-bib-0035] Perez, J. , Buchanan, A. , Mellbye, B. , Ferrell, R. , Chang, J. H. , Chaplen, F. , … Sayavedra‐Soto, L. A. (2015). Interactions of *Nitrosomonas europaea* and *Nitrobacter winogradskyi* grown in co‐culture. Archives of Microbiology, 197, 79–89. 25362506 10.1007/s00203-014-1056-1

[mbo3559-bib-0502] Reasoner, D. J. , & Geldreich, E. E . (1985). A new medium for the enumeration and subculture of bacteria from potable water. Applied and Environmental Microbiology, 49, 1–7. 3883894 10.1128/aem.49.1.1-7.1985 PMC238333

[mbo3559-bib-0037] Rickard, A. H. , Palmer, R. J., Jr. , Blehert, D. S. , Campagna, S. R. , Semmelhack, M. F. , Egland, P. G. , … Kolenbrander, P. E. (2006). Autoinducer 2: A concentration‐dependent signal for mutualistic bacterial biofilm growth. Molecular Microbiology, 60, 1446–1456. 16796680 10.1111/j.1365-2958.2006.05202.x

[mbo3559-bib-0038] Rosenthal, A. Z. , Matson, E. G. , Eldar, A. , & Leadbetter, J. R. (2011). RNA‐seq reveals cooperative metabolic interactions between two termite‐gut spirochete species in co‐culture. ISME Journal, 5, 1133–1142. 21326336 10.1038/ismej.2011.3 PMC3146290

[mbo3559-bib-0039] Russell, A. B. , Peterson, S. B. , & Mougous, J. D. (2014). Type VI secretion system effectors: Poisons with a purpose. Nature Reviews Microbiology, 12, 137–148. 24384601 10.1038/nrmicro3185 PMC4256078

[mbo3559-bib-0040] Sabag‐Daigle, A. , Dyszel, J. L. , Gonzalez, J. F. , Ali, M. M. , & Ahmer, B. M. (2015). Identification of *sdiA*‐regulated genes in a mouse commensal strain of *Enterobacter cloacae* . Frontiers in Cellular and Infection Microbiology, 5, 47. 26075189 10.3389/fcimb.2015.00047 PMC4444967

[mbo3559-bib-0041] Sambrook, J. , & Russell, D. W. (2001). Molecular cloning: A laboratory manual. Cold Spring Harbor, NY: Cold Spring Harbor Laboratory Press.

[mbo3559-bib-0042] Shimoyama, T. , Kato, S. , Ishii, S. , & Watanabe, K. (2009). Flagellum mediates symbiosis. Science, 323, 1574. 19299611 10.1126/science.1170086

[mbo3559-bib-0043] Subramoni, S. , & Venturi, V. (2009). LuxR‐family “solos”: Bachelor sensors/regulators of signalling molecules. Microbiology, 155, 1377–1385. 19383698 10.1099/mic.0.026849-0

[mbo3559-bib-0044] Suzuki, S. , Horinouchi, T. , & Furusawa, C. (2014). Prediction of antibiotic resistance by gene expression profiles. Nature Communications, 5, 5792. 10.1038/ncomms6792 PMC4351646 25517437

[mbo3559-bib-0045] Tai, V. , Paulsen, I. T. , Phillippy, K. , Johnson, D. A. , & Palenik, B. (2009). Whole‐genome microarray analyses of *Synechococcus‐Vibrio* interactions. Environmental Microbiology, 11, 2698–2709. 19659554 10.1111/j.1462-2920.2009.01997.x

[mbo3559-bib-0046] Teramoto, J. , Yamanishi, Y. , el Magdy, S. H. , Hasegawa, A. , Kori, A. , Nakajima, M. , … Ishihama, A. (2010). Single live‐bacterial cell assay of promoter activity and regulation. Genes to Cells, 15, 1111–1122. 20964794 10.1111/j.1365-2443.2010.01449.x

[mbo3559-bib-0048] Ward, C. B. , & Glaser, D. A. (1971). Correlation between rate of cell growth and rate of DNA synthesis in *Escherichia coli* B‐r. Proceedings of the National Academy of Sciences of the United States of America, 68, 1061–1064. 4930241 10.1073/pnas.68.5.1061 PMC389113

[mbo3559-bib-0049] Wenter, R. , Hutz, K. , Dibbern, D. , Li, T. , Reisinger, V. , Ploscher, M. , … Overmann, J. (2010). Expression‐based identification of genetic determinants of the bacterial symbiosis *Chlorochromatium aggregatum* . Environmental Microbiology, 12, 2259–2276. 21966918 10.1111/j.1462-2920.2010.02206.x

[mbo3559-bib-0050] West, S. A. , Griffin, A. S. , & Gardner, A. (2007). Social semantics: Altruism, cooperation, mutualism, strong reciprocity and group selection. Journal of Evolutionary Biology, 20, 415–432. 17305808 10.1111/j.1420-9101.2006.01258.x

[mbo3559-bib-0051] Wu, Y. , Mohanty, A. , Chia, W. S. , & Cao, B. (2016). Influence of 3‐Chloroaniline on the biofilm lifestyle of *Comamonas testosteron*i and its implications on bioaugmentation. Applied and Environment Microbiology, 82, 4401–4409. 10.1128/AEM.00874-16 PMC4959187 27208104

[mbo3559-bib-0052] Zerbino, D. R. , & Birney, E. (2008). Velvet: Algorithms for de novo short read assembly using de Bruijn graphs. Genome Research, 18, 821–829. 18349386 10.1101/gr.074492.107 PMC2336801

[mbo3559-bib-0053] Zgur‐Bertok, D. (2013). DNA damage repair and bacterial pathogens. PLoS Pathogens, 9, e1003711. 24244154 10.1371/journal.ppat.1003711 PMC3820712

[mbo3559-bib-0054] Zhou, Y. , Liang, Y. , Lynch, K. H. , Dennis, J. J. , & Wishart, D. S. (2011). PHAST: A fast phage search tool. Nucleic Acids Research, 39, W347–W352. 21672955 10.1093/nar/gkr485 PMC3125810

