# Supplementary figures and images for: Molecular processes underlying synergistic linuron mineralization in a triple‐species bacterial consortium biofilm revealed by differential transcriptomics
Source: Microbiologyopen. 2018 Jan 3;7(2):e00559. doi: 10.1002/mbo3.559 (PMC5911999; doi:10.1002/mbo3.559)

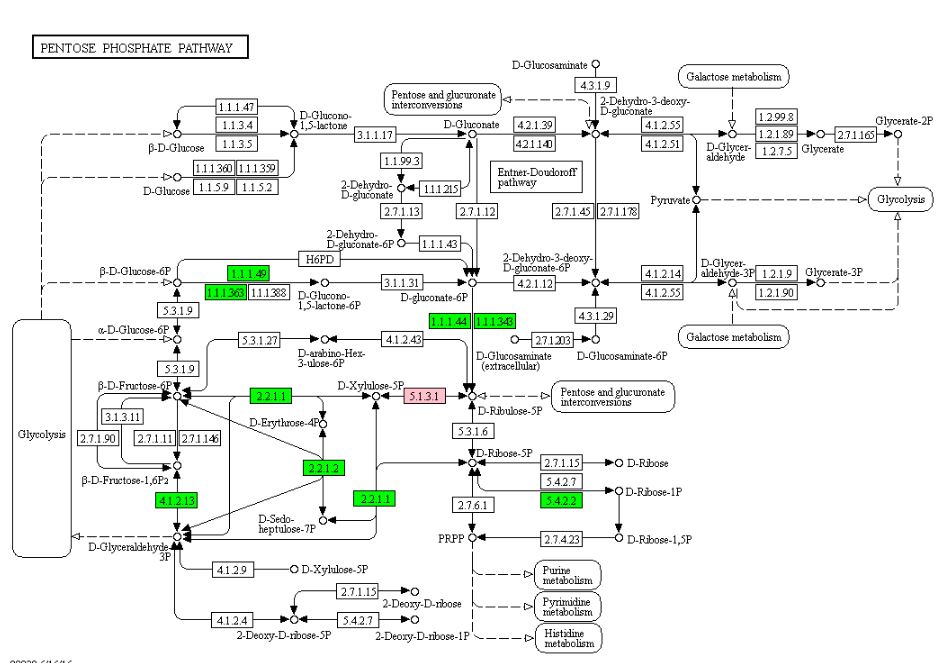

Supplement: Supplementary file 2 [file MBO3-7-na-s002.jpg]

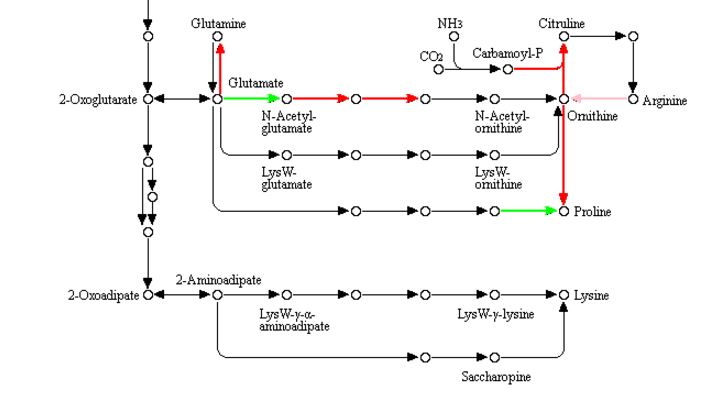

Supplement: Supplementary file 3 [file MBO3-7-na-s003.jpg]

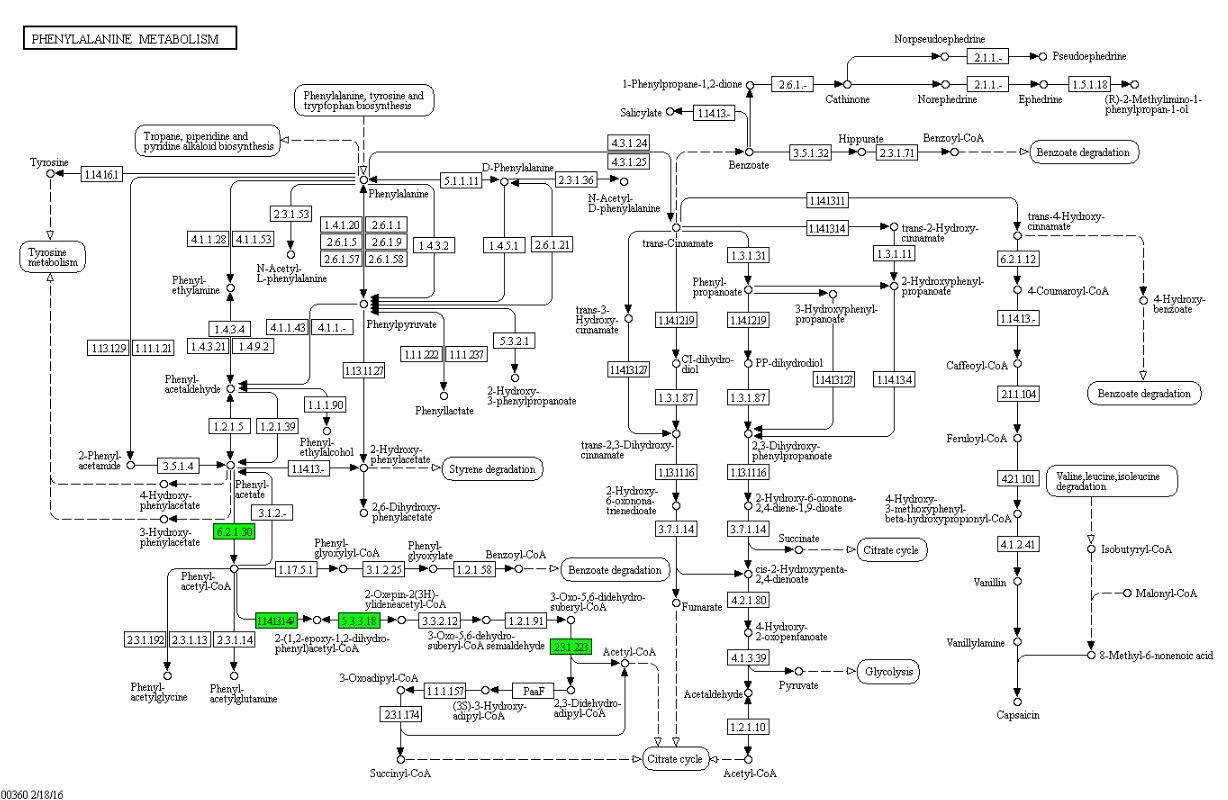

Supplement: Supplementary file 4 [file MBO3-7-na-s004.jpg]

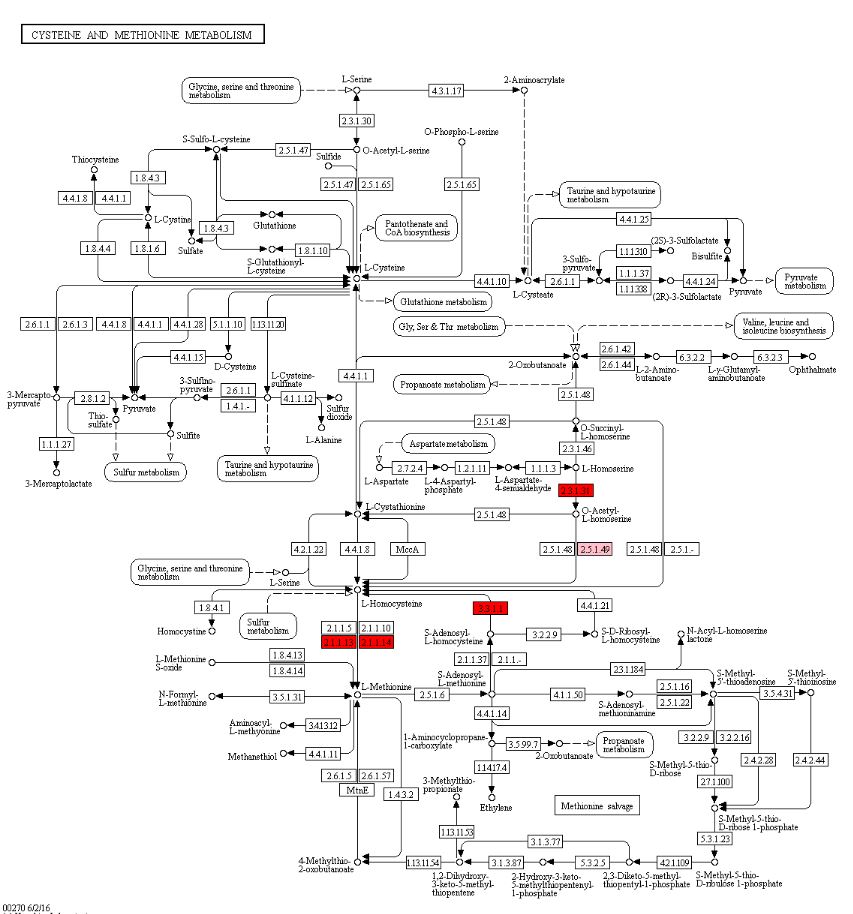

Supplement: Supplementary file 5 [file MBO3-7-na-s005.jpg]

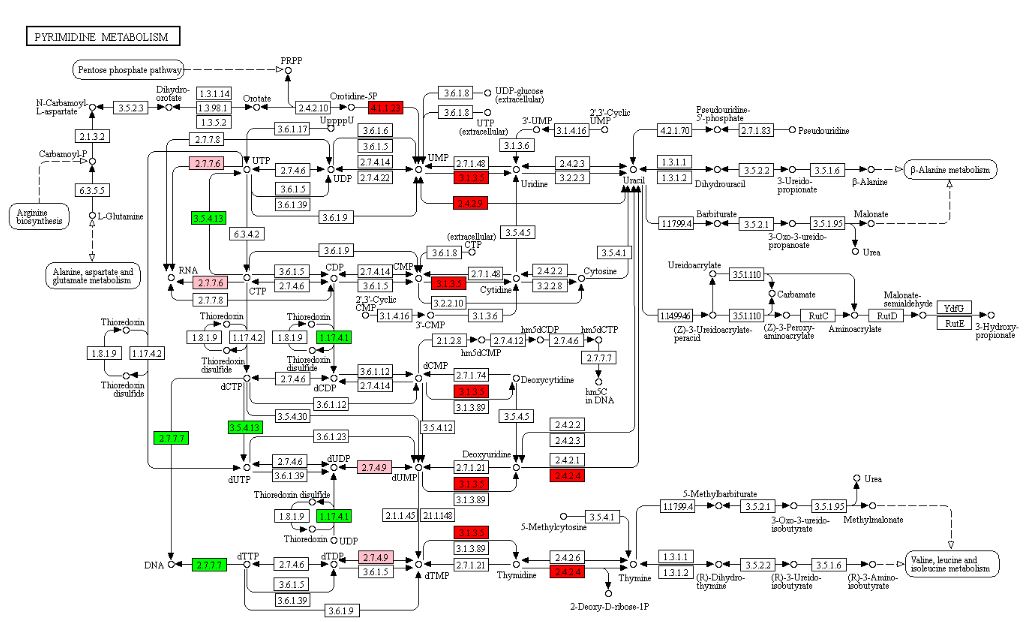

Supplement: Supplementary file 6 [file MBO3-7-na-s006.jpg]

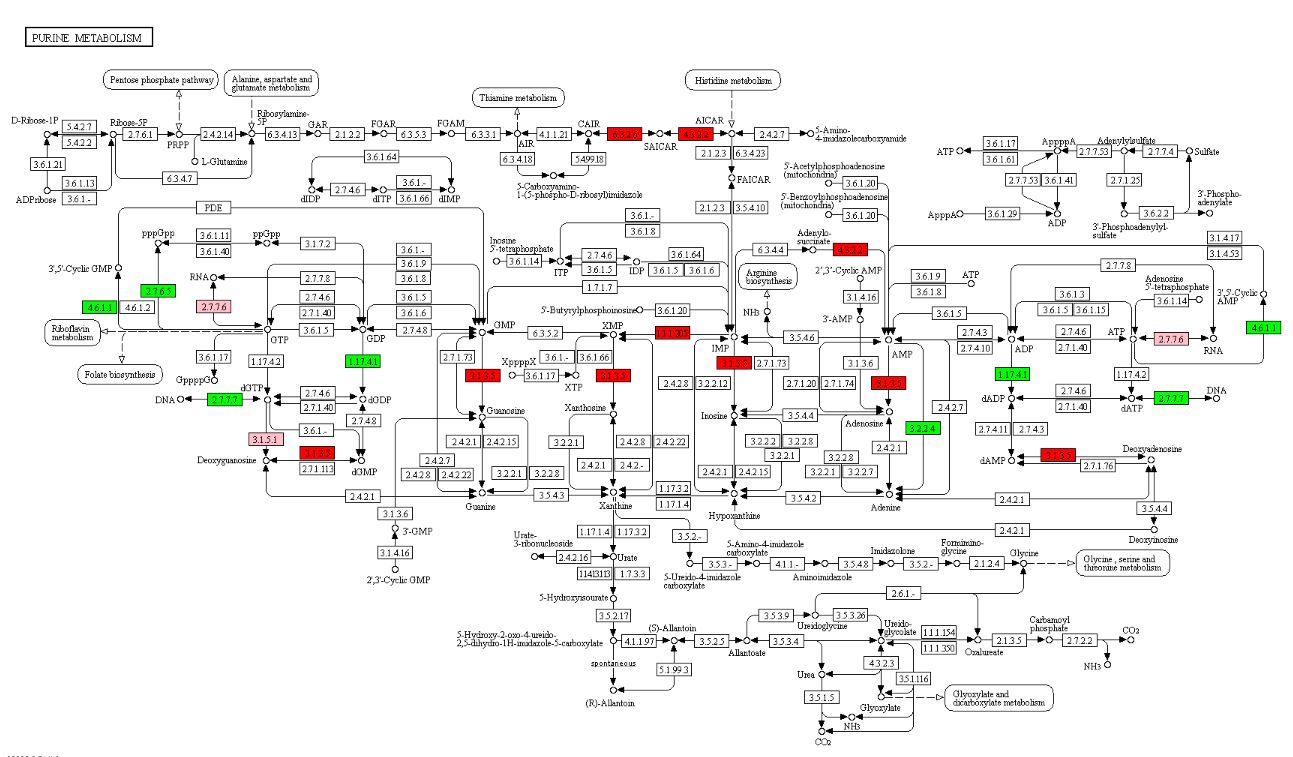

Supplement: Supplementary file 7 [file MBO3-7-na-s007.jpg]

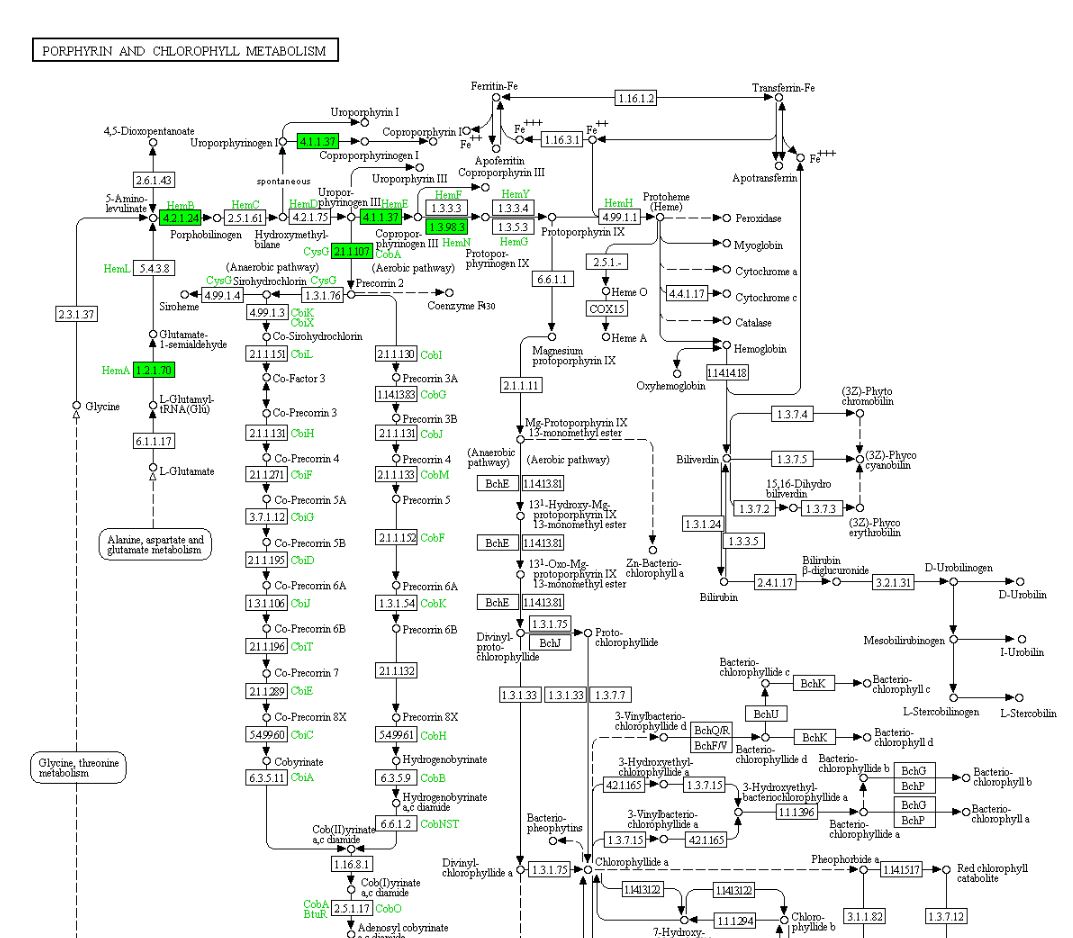

Supplement: Supplementary file 8 [file MBO3-7-na-s008.jpg]

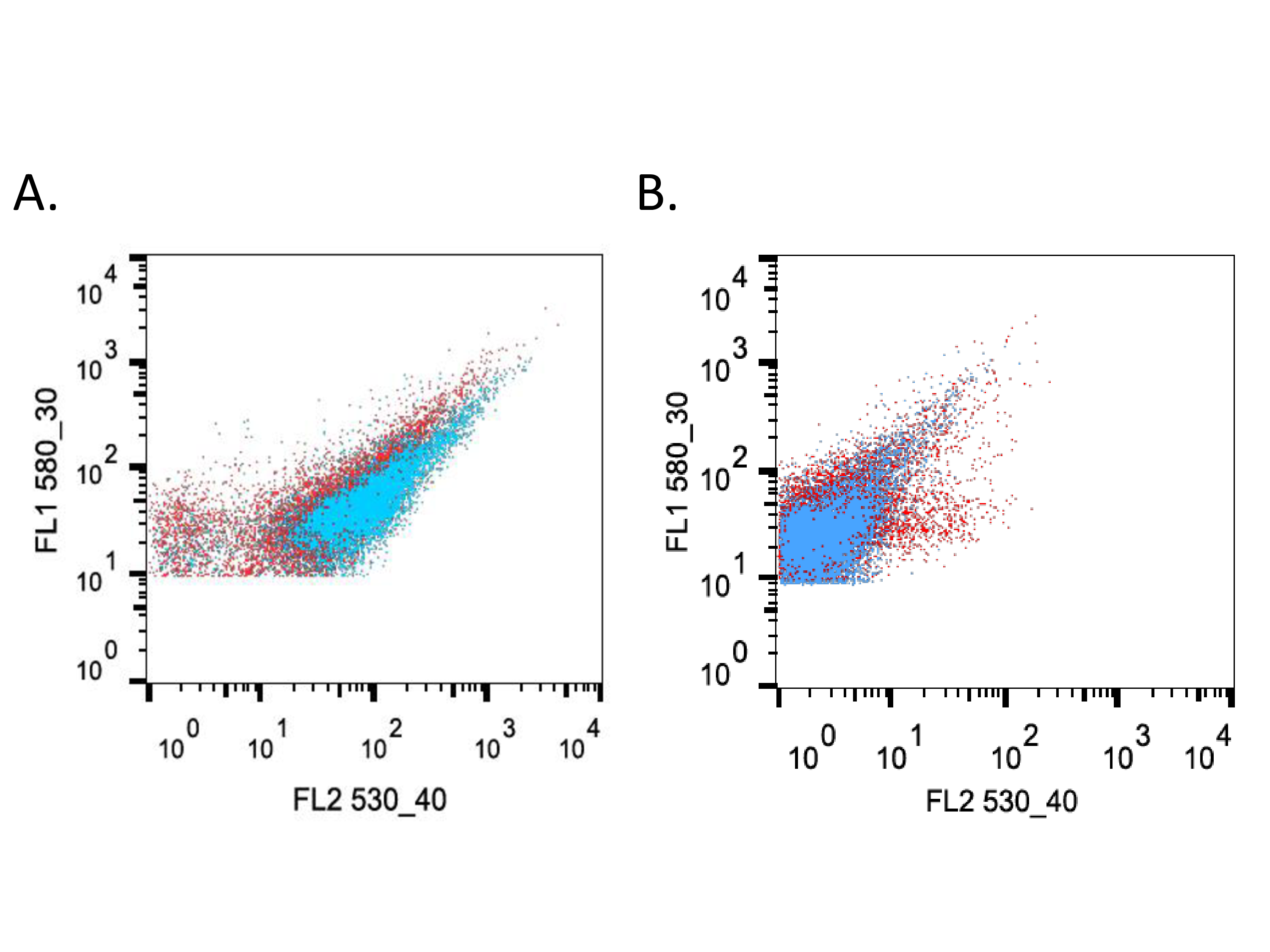

Supplement: Supplementary file 9 [file MBO3-7-na-s009.tif]
