# Supplementary material for: Molecular processes underlying synergistic linuron mineralization in a triple‐species bacterial consortium biofilm revealed by differential transcriptomics
Source: Microbiologyopen. 2018 Jan 3;7(2):e00559. doi: 10.1002/mbo3.559 (PMC5911999; doi:10.1002/mbo3.559)
Supplement: Supplementary file 11 [file MBO3-7-na-s011.docx]

# Supporting tables S2-S3-S5

**Table S2.** Fractions (in %) of read pairs that mapped on linuron catabolic genes in WDL7 and WDL1 of total read pairs mapping with CDSs

|  | WDL1 | |  | | |  | | WDL7 | |  |
| --- | --- | --- | --- | --- | --- | --- | --- | --- | --- | --- |
| Catabolic cluster | Consortium | Monoculture | |  | Catabolic cluster | | Consortium | | Monoculture | |
| *hylA* | 16.7% | 0.2% | |  |  | |  | |  | |
| *dca* | 11.5% | 11.7% | |  | *dca* | | 16.0% | | 20.1% | |
| *ccd* | 3.7% | 6.2% | |  | *catAB, CMBL, tfdF* | | 9.6% | | 9.2% | |
| *pca* | 0.1% | 0.4% | |  | *pca* | | 0.7% | | 1.7% | |
| Sum | 32.2% | 18.5% | |  | Sum | | 26.3% | | 31.0% | |

**Table S3**. Summary of draft genome sequencing results. *PExx = Paired-end sequencing followed by a number that refers to the read length.

|  | **WDL1** | **WDL6** | **WDL7** |
| --- | --- | --- | --- |
| N50 | 76000 | 94254 | 106400 |
| Contigs>100bp | 493 | 109 | 151 |
| Coverage | 24 | 44 | 45 |
| Genome size (bp) | 8,170,112 | 3,705,164 | 5,541,122 |
| GC content (%) | 66.4 | 61.1 | 61.5 |
| Number of CDSs | 7759 | 3496 | 5069 |
| Sequencing details* | PE 75 | PE90 | PE75 |
| Total data size | 575 MB | 418 MB | 751 MB |

**Table S4.** Calculated scaling factors for genes of WDL1 and WDL7

**Table S5.** Primers used in this study

| **Target** | **Genbank ID** | **purpose** | **Primer sequence (5’-3’)** | **Reference** |  |
| --- | --- | --- | --- | --- | --- |
| *omp32*  in WDL1 | APY03_1563 | conventional | ACCTGCTTGGCGTGACCTG | this study |  |
|  |  |  | AGGCCAGCGCCGTTCTTGTT |  |  |
|  |  | real time | GCCTGATCCGCGCGTCCTAC |  |  |
|  |  |  | GCCGTGCGCTTCGACAGGTT |  |  |
| *hylA*  in WDL1 | APY03_6463 | conventional | AGGTCATGTCCACTCGCGTCT | Horemans *et al.*, 2016 |  |
|  |  |  | GCCGATGCATAGGGCCATATTTGCT |  |  |
|  |  | real time | GCATGGGTCTGTTGCTGATAC |  |  |
|  |  |  | CTGCGTGGAACTTCACTGTTAG |  |  |
| *catA*  in WDL1 | AGF25473.1 | conventional | ACGCCACCATCACTGACAAT | this study |  |
|  |  |  | CCCATCATCTCGAGCAGTT |  |  |
|  |  | real time | CACTCGGACAATCACGGACA |  |  |
|  |  |  | GTACTCACCGTCTGCACCAA |  |  |
| *phoA*  in WDL1 | APY03_7531 | conventional | GGCGGCAACGACATCCTGCT | this study |  |
|  |  |  | GCCGACCTGTGCGTTGGTCA |  |  |
|  | KWT65078.1 | real time | ACGCTATTTGGCGGAGGCGG |  |  |
|  |  |  | CCGGCCAATGAGGTGGTGGG |  |  |
| *dcaQ*  in WDL1 | AGF25498.1 | conventional | TGCTGGCCGACCTTTACATGA | this study |  |
|  |  |  | CTACAAAGCCTTGGATGGCGG |  |  |
|  |  | real time | GTACCTGCTGGAGAACCACCT |  |  |
|  |  |  | GGAACAGCACCATCGTGTCC |  |  |
| *rpoA*  in WDL7 | APV28_2044 | conventional | GCTTGACGGGCGAGAACGAA | this study |  |
|  |  |  | TTCTGGAGCCGTTTGAGCGTGG |  |  |
|  |  | real time | GGTTCAGCAGGATGTTGGTC |  |  |
|  |  |  | CGTGTTCTGCTCTCCTCCA |  |  |
| *pcaF*  in WDL7 | APV28_0875 | conventional | AGCTTAACGAAGCCTTTGCC | this study |  |
|  |  |  | GCGAGACAGGGAAAGGGAAT |  |  |
|  | KWT73476.1 | real time | TGAACCAGCTGCATGCCTT |  |  |
|  |  |  | TTCGAGCACCACGGCAAT |  |  |
| *yrbC*  in WDL7 | APV28_0860 | conventional | GATGTGCTGGAGACCATCAA | this study |  |
|  |  |  | TGACGGTAATGGTCTGATCG |  |  |
|  | KWT73461.1 | real time | ATGCCCTATGTGGACTTTCG |  |  |
|  |  |  | ATAGGTGCGAATCAGCAAGG |  |  |
| *pilM*  in WDL7 | APV28_0896 | conventional | CGGAAGAGGCAGAAGCTAAG | this study |  |
|  |  |  | GAAAGGATTGGCAAGACTGC |  |  |
|  | KWT73497.1 | real time | CGAATTGCCAGACGACTATG |  |  |
|  |  |  | ATGATCGACGCGGTTATAGG |  |  |
| *pilY1*  in WDL7 | APV28_0438 | conventional | CCCAGCAACAAAGTTCCATT | this study |  |
|  |  |  | TGATCTTGCCAATGATTTCG |  |  |
|  | KWT74311.1 | real time | TCTCCAGCGCACATCATAAG |  |  |
|  |  |  | CGAAATGCTTTGGCTAGGTC |  |  |
| *glxR*  in WDL7 | APV28_4200 | conventional | AGATGGGCAAGAACATCACC | this study |  |
|  |  |  | GATCGAAGGTGCGCTTGA |  |  |
|  | KWT66982.1 | real time | TCATCGTGGCTCTGAACATC |  |  |
|  |  |  | GGATCTTGGAAGAGGCAAAG |  |  |
| WDL1 16S rRNA gene | | APY03_7152 | conventional | CAATCGTGGGGGATAACGC | Bers *et al.*, 2011 |
|  |  |  |  | ATTACCGCGGCTGCTGG |  |
| WDL7 16S rRNA gene | | APV28_0001 | conventional | TAGTGGGGGATAACTACTCG | this study |
|  |  |  |  | CATGACCCGGGGATATTAGC |  |
| *gcl* promoter in WDL7 |  | conventional | AGCAAGCTTACCTGCGGGTTGTGCTTCAT | this study |  |
|  |  |  | CGAGGATCCTGCGGCTTCGATTGCTTTCA |  |  |
| *pca* promoter in WDL7 |  | conventional | AGCAAGCTTCCGAGCATGACTCCTTGACC | this study |  |
|  |  |  | CGAGGATCCCGAGTCGGCGATCTTGTTGA |  |  |
