# Supplementary material for: Molecular processes underlying synergistic linuron mineralization in a triple‐species bacterial consortium biofilm revealed by differential transcriptomics
Source: Microbiologyopen. 2018 Jan 3;7(2):e00559. doi: 10.1002/mbo3.559 (PMC5911999; doi:10.1002/mbo3.559)
Supplement: Supplementary file 13 [file MBO3-7-na-s013.pdf]

Supporting Experimental Procedures

Conventional PCR

Amplification of standard template for real time quantitative PCR assays was performed using the conventional PCR primer sets listed in Table S5. Primers were designed using Primer3-v.0.4.0 software (Untergasser et al., 2012). PCR reactions were performed in a total volume of 25 µL containing 2.5 µL 10x Dream Taq Green buffer (Thermo Scientific, USA), 2 µL 2.5 mM dNTPs, 0.125 µL of both primers, 0.125 µL DreamTaq DNA Polymerase (5 U/µL) (Thermo Scientific, USA) and 1 µL of template DNA. PCR reactions were performed in a Biometra Thermocycler (AnalytikJena, Germany) and consisted of an initial denaturation step of 5 min at 94°C, followed by 35 cycles of 30 sec at 94°C, 20 sec at 60°C and 20 sec at 72°C and a final elongation step at 72°C for 5 min Amplicons were visualized by agarose gel electrophoresis (1% agarose, 60 min, 90V) using Gelred (Biotum, USA) as nucleic acid stain.

Real time reverse transcription quantitative PCR

One ng of rRNA-depleted RNA was used for first strand cDNA synthesis, using the RevertAid H Minus First strand cDNA synthesis kit (Thermo Scientific™), according to the manufacturer's instructions. A 1:5 dilution of resulting cDNA was used as a template for qPCR. For all qPCR reactions, the reaction mixture contained 7.5 µL of Absolute QPCR SYBRs Green mix (Thermo Scientific, USA), 0.03 µL of both forward and reverse primer (100 µM), 5.94 µL of water, and 1.5 µL of diluted cDNA. Primers were designed using Primer3-v.0.4.0 software (Untergasser et al., 2012) and beforehand tested for the desired specificity on genomic DNA of strains WDL1, WDL7 and WDL6. Primers are listed in Table S5. qPCR reactions were performed in duplicate in a Rotor

Gene 6000 real-time cycler apparatus (Qiagen, Germany). The qPCR reaction consisted of an initial denaturation step of 15 min at 94°C, followed by 40 cycles of 20 s at 94°C, 20 s at 60°C and 20 s at 72°C. To generate a standard curve, a 10-fold dilution series of 10^8 copies μL^{-1} of amplicons of appropriate gene fragments generated by conventional PCR on genomic DNA of WDL1 or WDL7, was included in every qPCR assay. The amplicons were purified from agarose gels using the QIAquick Gel Extraction Kit (Qiagen, Germany) and DNA concentrations of the purified DNA fragments were determined with the NanoDrop 1000 Spectrophotometer (Thermo Scientific, USA). The detection limit of all qPCR assays was 10 copies μL^{-1}. The transcript number of *omp32* from WDL1 and *rpoA* from WDL7 were used to normalize the copy number of target gene transcripts to the amount of WDL1- or WDL7-specific cDNA in monoculture and consortium samples since both *omp32* and *rpoA* showed no differential expression between monoculture and multispecies conditions based on RNAseq data. For all samples, relative gene expression values were calculated as the log2 ratio of the number of target gene transcripts to the number of transcripts of either *omp32* and *rpoA*. qPCR-based log2-fold change of gene expression between monoculture and consortium conditions was calculated by subtracting the average relative gene expression value in monoculture conditions from the average relative gene expression value in consortium conditions.

Verification of differential transcription using transcriptional gene fusions

For molecular cloning, plasmid isolation and *E. coli* competent cell preparation and transformation, standard procedures were used (Sambrook and Russell, 2001). The 500 bp promoter region of the glycerate biosynthesis operon (*gcl*) of WDL7 (P_{gcl}) was PCR amplified with forward primer (AGCAAGCTTACCTGCGGGTTGTGCTTCAT) and reverse primer

(CGAGGATCCTGCGGCTTCGATTGCTTTCA). The 494 bp promoter region of the 3-oxoadipate catabolic operon (*pca*) of WDL7 (P_{pca}) was PCR amplified with forward primer (AGCAAGCTTCCGAGCATGACTCCTTGACC) and reverse primer (CGAGGATCCCGAGTCGGCGATCTTGTTGA). For both PCRs, WDL7 genomic DNA was used as a template and both forward and reverse primers contained respectively a HindIII and BamHI site allowing directional cloning. The PCR amplified promoter fragments were digested with HindIII and BamHI (Thermo Fischer Scientific) and ligated into the multiple cloning site of pRU1097 in front of a promoterless *gfpmut3.1* (Karunakaran et al., 2005) using T4 DNA ligase (Thermo Fischer Scientific) creating plasmid pSWPA1 containing the *pca* promotor region and pSWPA2 containing the *gcl* promotor region. Plasmids were electroporated into TransforMax™ EC100™ *E. coli* cells (Epicentre) using a MicroPulser (Bio-Rad). The insert sequence was verified by Sanger sequencing. pSWPA1 and pSWPA2 were conjugated into WDL7-RFP, a variant of WDL7 constitutively expressing the Rfp protein (Breugelmans et al., 2008) by triparental mating with *E. coli* DH5α (pRK2013) as a helper strain and transconjugants were selected on MMO supplemented with 200 mg L^{-1} aniline and 140 mg L^{-1} gentamicin and 50 mg mL^{-1} kanamycin. The presence of the reporter plasmids in the transconjugants was tested with colony PCR. Monocultures of WDL7-Rfp with pSWPA1/pSWPA2 and consortium cultures containing WDL7-Rfp with pSWPA1/pSWPA2 were inoculated in flow channels in triplicate and grown as biofilms continuously fed with MMO supplemented with either 3,4-DCA or linuron as described above. After two weeks of steady-state conditions, biofilms cells were flushed out of the flow channels using 1 mL 10 mM $MgSO_4$ as described above. Cell suspensions were analyzed using a BD Influx™ (BD Biosciences) cell sorter calibrated with Sphero™ Rainbow Calibration particles

(Spherotech). Instrument settings were empirically optimized using WDL7-Rfp carrying the pRU1097 vector without insert as a negative control. Fluorescence, side scatter, forward scatter and pulse width data were collected for 10^5 cells using logarithmic amplifiers. WDL7-Rfp cells showing constitutive *rfp* expression were distinguished from other particles (cell aggregates, fragments, debris and non-fluorescent cells) by setting pulse width, forward scatter and red fluorescence (excitation 488 nm, emission filter FL01 580/30) as a primary, secondary and third threshold, respectively. The data were analyzed using FlowJo (Tree Star Inc., Ashland, OR). For each sample, the mean GFP expression (excitation 488 nm, emission filter FL02 540/30) of WDL7-Rfp variants carrying pSWPA1 or pSWPA2 was calculated and significant differences between monoculture and consortium conditions were determined using a one tailed Student t-test ($P < 0.05$).
